# Documenting archaeological thin sections in high‐resolution: A comparison of methods and discussion of applications

**DOI:** 10.1002/gea.21706

**Published:** 2018-11-13

**Authors:** Magnus M. Haaland, Matthias Czechowski, Frank Carpentier, Mathieu Lejay, Bruno Vandermeulen

**Affiliations:** ^1^ Institute for Archaeology, History, Culture and Religious Studies, Faculty of Humanities, University of Bergen Bergen Norway; ^2^ Centre for Early Sapiens Behaviour (SapienCE) University of Bergen Bergen Norway; ^3^ Institute for Archaeological Sciences, Department of Geosciences, University of Tübingen Tübingen Germany; ^4^ Senckenberg Center for Human Evolution and Palaeoenvironment, University of Tübingen Tübingen Germany; ^5^ Department of Archaeology KU Leuven Leuven Belgium; ^6^ TRACES Laboratory–UMR 5608, CNRS, University of Toulouse II Jean Jaurès Toulouse France; ^7^ ULS Digitisation and Document Delivery, KU Leuven Leuven Belgium

**Keywords:** micromorphology, archiving, digital documentation, digital thin sections, visual communication

## Abstract

Optical thin section observations represent the core empirical basis for most micromorphological interpretations at archaeological sites. These observations, which often vary in size and shape, are usually documented through digital graphic representations such as photomicrographs, scans, or figures. Due to variability in documentation practices, however, visual thin section data can be captured with a range of methods and in many different formats and resolutions. In this paper, we compare and evaluate five common image‐based methods for documenting thin sections in high‐resolution: a flatbed scanner, a film scanner, a macro photography rig, and conventional stereo and light microscopes. Through the comparison results, we demonstrate that advances in digital imaging technology now allow for fast and high‐resolution visual recording of entire thin sections up to at least ×30 magnification. We suggest that adopting a digital micromorphological documentation practice has several advantages. First, a digital thin section may be observed more efficiently and consistently, for example, on a computer screen, and the spatial configuration of large or complex features may be more accurately documented. Second, they allow for the establishment of digital repositories that may promote scientific reproducibility and inter‐laboratory communication, as well as lay the foundations for more consensus‐based educational training of archaeological micromorphology.

## 1 INTRODUCTION

By its very nature, conventional archaeological micromorphological analysis is highly qualitative. This is not only due to the selective sampling procedures conducted in the field, which is affected by a range of informed but subjective choices (Courty, Goldberg, & Macphail, 1989; Nicosia & Stoops, 2017), but also due to the fact that most thin section observations and interpretations are governed by the practitioners’ own experience, individual examination procedures, and personal documentation skills (Goldberg & Aldeias, 2018). The fact that archaeological micromorphology involves a certain degree of subjectivity has previously been discussed by Shahack‐Gross (2016), who through an intracommunity self‐evaluation exercise, demonstrated that the accuracy of basic material identification in thin sections could vary a lot from person to person, and that successful material identification relied heavily on the researcher's academic training and experience. Shahack‐Gross (2016) argues that there are several ways to improve the accuracy of micromorphological data production and to avoid erroneous interpretations, such as holding regular micromorphological working group meetings and workshops and routinely conducting self‐evaluation tests to identify analytical blind spots. In addition to these now established practices, we would argue that because optical thin section observations are such an integral part of the overall analytical process, archaeological micromorphologists should support their published papers and interpretations, not only with individual photomicrographs, but also with complete and detailed graphical datasets of entire thin sections. Such high‐quality, high‐resolution thin section documentation would effectively allow anyone to independently examine and evaluate these thin sections in the most relevant resolution, scale, or light setting.

Currently, there are two conventional ways of documenting a thin section: (a) by written text or numbers, or (b) by visual documentation, that is images. The written part typically involves making detailed descriptions or schematic overviews, such as tables or charts, which summarize the qualitative observations most often made with the aid of conventional petrographic light microscopes. While standardized protocols for written micromorphological descriptions and terminology have been put forward (Brewer, 1976; Bullock, Fedoroff, Jongerius, Stoops, & Tursina, 1985; Courty et al., 1989; Goldberg & Macphail, 2006; Macphail & Goldberg, 2010; Nicosia & Stoops, 2017; Stoops, 2003), few guidelines exist for the visual documentation of archaeological thin sections. This may relate to the fact that archaeological sites are incredibly variable and complex, and rigid documentation protocols would simply not be practical for describing such a diversity of contexts.

Often, the only feasible way to characterize accurately, communicate, and fully appreciate the complexity seen in many archaeological thin sections is through digital image‐based documentation. Consequently, the visual documentation of thin sections, for example photomicrographs, should not be regarded as basic illustrations when in fact they represent the core empirical basis for most micromorphological interpretations. According to Goldberg and Aldeias (2018), the analytical and evaluative power of visual thin section documentation should not be underestimated, and should rather be regarded as one of the most significant analytical outputs from any micromorphological investigation of archaeological deposits.

Due to the considerable variability in laboratory infrastructure, a wide range of image‐based thin section recording procedures currently exist, resulting in datasets captured in different formats and resolutions, at different scales and taken with various light settings. The lack of consistent visual documentation standards does not only make it difficult to autonomously evaluate published image‐based interpretations, but can also make it exceedingly difficult to compare thin section observations from one context to another. Furthermore, a limiting trait of most thin section documentation has always been the dynamic relationship between data resolution and field of view (De Keyser, 1999). Most microscope image‐capturing systems typically allow for either low‐magnification documentation of larger areas or high‐magnification documentation of smaller areas. In thin sections made from archaeological block samples, however, many important microstratigraphic relationships often extend beyond the restricted field of view of a high‐magnification microscope; and simultaneously the very same microscopic relationships cannot be accurately observed or mapped using only low‐magnification, wide‐angle image‐capturing settings.

The issue of analytical scale has also been debated by Goldberg and Aldeias (2018), who have argued that one of the main reasons why archaeological micromorphology is by nonspecialists still regarded as a somewhat unapproachable technique is due to the often poor integration of microscopic thin section observations with general macroscopic field data. Therefore, to make archaeological micromorphology more accessible to a broader audience, Goldberg and Aldeias (2018) call for the adoption of so‐called *mesoscopic* analytical approaches that can visually bridge the analytical gap between macroscale and microscale geoarchaeological site investigations. That is, methods and approaches capable of linking observations made in the field with the naked eye to observations made in a lab at successively higher magnifications, for example through a microscope. Despite its obvious analytical advantages there are, however, surprisingly few examples of systematic and successful multiscalar integration of micromorphological and macroscopic field data (but see Fisher et al., 2015; Karkanas, Brown, Fisher, Jacobs, & Marean, 2015, for pionering work).

**Figure 1:**
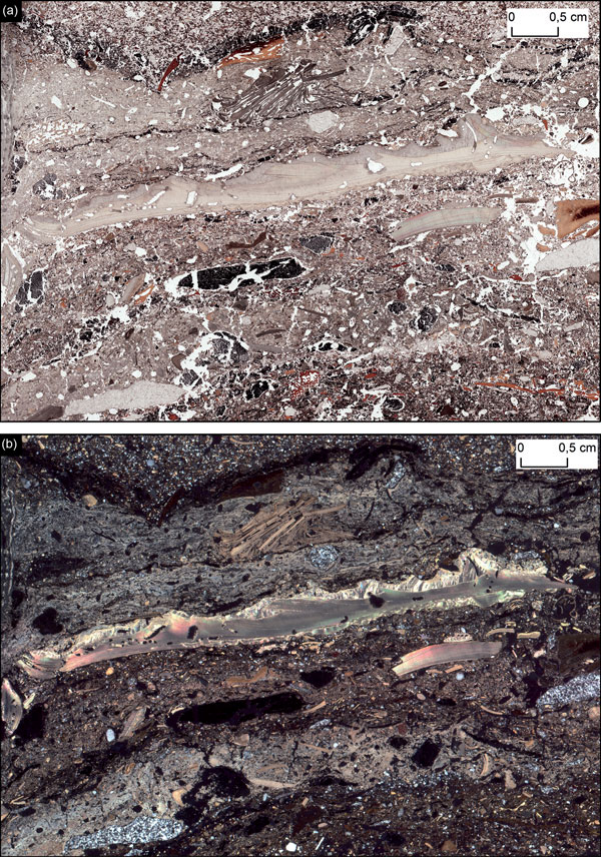
The thin‐section used for visual reference in this study in (a) PPL and (b) XPL (4,000 DPI film‐scan). Note the complex microstratigraphy which contains numerous bone fragments, shellfish, charcoal, humified, and charred organic material, ash, lithic debris (quartzite), and quartz‐rich sand. The sample is taken from the Middle Stone Age site of Klipdrift Shelter, South–Africa from an archaeological horizon dated to 65,000 years ago. See Supporting Information Figures A.1–A.10 for high‐resolution images of this thin section made with different methods (flatbed scanner, film scanner, DSLR macro photography, and motorized stereo zoom microscope) in different light settings (PPL, XPL, and reflective light). DPI: dots per inch; DSLR: digital single lens reflex; PPL: plane‐polarized light; XPL: cross‐polarized light [Color figure can be viewed at wileyonlinelibrary.com]

Due to the development of digital imaging technology and the increase in computer power, there are multiple documentation methods that exist today and are capable of seamlessly capturing and displaying entire thin sections in both high and low magnification. While several of these methods have previously been discussed (Arpin, Mallol, & Goldberg, 2002; Carpentier & Vandermeulen, 2016; De Keyser, 1999), there have been no systematic comparisons or actualistic assessments of them. In this paper, therefore, we evaluate and compare the most commonly used methods for visual thin section documentation (flatbed scanner, film scanner, macro photography, stereo zoom microscope, and polarized light microscope) by applying each method to a reference thin section (Figure 1). Based on this comparison, we then discuss each method's practical and analytical advantages and limits, before discussing how new and digitized practices can greatly improve how archaeological micromorphologists analyze their samples, and how they communicate their results and interpretations.

## 2 BACKGROUND TO METHODS AND COMPARISON PARAMETERS

The most common way to document petrographic thin sections is by photographs captured through a microscope setup (i.e., photomicrographs). These setups typically contain a camera system that connects to the microscope, most often via a dedicated phototube on a trinocular head. The resolution of the captured photos is determined by the image sensor of the camera involved and the field of view by the eyepiece diaphragm opening diameter (at any given magnification). Due to the limited field of view of most microscope setups, photomicrographs are typically used to document single features or spatially confined areas within a single thin section. To achieve greater areal documentation, some microscope software solutions allow you to capture multiple, overlapping images and then automatically stitch these together to form a larger photomosaic. A similar panoramic effect is also achievable by importing the overlapping images into third‐party stitching software. However, the creation of high‐resolution, seamless photomosaics from multiple microscope photographs can become a rather time‐consuming process, especially if the capturing procedure requires a high amount of manual involvement from the user. Furthermore, photo‐capturing inconsistency caused by changes in light conditions, white balance, focus plane, or even the evenness and quality of the thin sections themselves, may also affect the quality of the final image output.

To overcome the limited field‐of‐view in microscope‐based visual documentation and to counter the capturing inconsistencies inherent in many photo‐stitching solutions, alternative image‐based documentation techniques capable of documenting entire thin sections in high resolution have been developed. These include the use of commercially available flatbed scanners (Arpin et al., 2002), medium‐format film scanners (De Keyser, 1999; Tarquini & Favalli, 2010) and macro photography solutions using digital single lens reflex (DSLR) cameras mounted with a macro lens (Carpentier & Vandermeulen, 2016). Whereas many flatbed scanners provide satisfactory results at low magnifications (× <10), only film scanning and macro photography have been reported to provide results comparable in resolution and quality to those of individual microphotographs (×30). Our goal in this paper, however, is not to declare a single method superior, but rather to understand how each of these methods offers specific advantages and can be used to complement or overlap one another.

Considering the great acquisition cost of transmitted light (TL) microscopes and stereoscopes, we also explore and compare alternative and less costly solutions, such as a consumer‐grade flatbed scanner, a professional film scanner, and a high‐resolution DSLR camera. In this regard, it is important to stress that merely reading the technical information provided by the equipment manufacturers is not sufficient for making a good practical comparison between different image‐based documentation methods. In some cases, technical information appears to be identical between consumer grade and professional grade equipment, whereas in reality there might be a huge difference in optical quality. This means that even if the technical output of different image‐capturing setups and technologies are the same, the actual image quality may vary.

The only way, therefore, to study and compare genuine differences between various recording methods, is to document the same thin section with all available methods and then compare and evaluate them through a fixed set of parameters. For this purpose, a particularly complex thin section made from an oriented block sample collected from the Middle Stone Age levels at Klipdrift Shelter, South Africa (Henshilwood et al., 2014) was selected as a visual reference (Figure 1 and Supporting Information Figures A.1–A.10). The deposits within these levels are characterized by a complex microstratigraphy which contains a variety of material types (e.g., bone, shellfish, ash, organic material, quartz, and quartzite), making them ideal for optical evaluations and comparisons. The selected block sample from Klipdrift Shelter was processed in the Geoarchaeology Laboratory at the University of Tübingen, Germany, where it first was dried at 40°C for 48 hr and then impregnated with resin under vacuum, in a 7:3 mixture ratio of unpromoted polyester resin (Viscovoss N 55S) and styrene, in addition to 5 ml/L hardener (MEKP). Once the block had hardened it was then cut to 6 × 9 × 3 cm chip, mounted on glass and subsequently ground and polished to a thickness of 30 μm.

In our assessment of the different thin section documentation techniques we focus on both technical and practical aspects of thin section image‐capturing, such as recording time, the general output formats (areal coverage, and resolution), the overall image quality (sharpness, resolution, and contrast) and available documentation settings (light settings and level of magnification). A full list of selected parameters is provided in Table 1. To describe the effective raster resolution (i.e., metric cell‐size value or pixel value) produced by each of the documentation methods tested, we calculate this in terms of microns per pixel (μm/pixel; similar to Tarquini & Favalli, 2010). Knowing how many microns each pixel or raster cell represents allows for a more intuitive understanding of how large a microscopic feature must be before it can be theoretically visible at various resolutions. It should here be mentioned that many hardware manufacturers often use *dots per inch* (DPI) or *pixels per inch* (PPI) to describe the reading resolution of their cameras or scanners. For example, the Nikon film scanner we tested for this paper (see below) is reported to have an *optical reading resolution* of 4,000 DPI or PPI (note that Nikon uses these terms interchangeably; Nikon, Inc., product web‐page accessed May 2018). To theoretically convert a 4,000 DPI/PPI raster value into a metric cell‐size value (μm/pixel) one may divide one inch (2.54 cm) by the PPI/ DPI value (i.e., 4,000), which would equal an effective raster resolution of around 6.35 microns (0.000635 cm) per cell or pixel. To be consistent with our actualistic approach, however, the metric cell‐size values (μm/pixel) we report in this paper were not only theoretically considered, but also independently calculated using GIS software (ArcGIS 10.5). We did this by first georeferencing each of the raster images (i.e., scans and photomosaics) to true scale and then evaluated the cell size of the newly georectified raster (as reported in the raster property information menu).

**Table 1:** Overview of relevant comparison parameters for image‐based thin section documentation

	Comparison parameters	Considerations/details
Equipment acquisition	Availability Cost Required infrastructure Maintenance level	Is the equipment/instrument easily available, including nonspecialist shops? Total cost of a functional documentation rig Additional infrastructure required for the rig to be operational Level of maintenance and service required
Installation and initial setup	First time installation Preparation complexity Preparation time Preparation cycle time	Difficulty level for first‐time installation Difficulty level for routine use and setup Time spent to make the equipment ready for recording Time spent per recording cycle
Practical use	Mobility Use with oil Range of uses	Can the documentation rig be easily moved? Can uncovered thin sections be easily treated with oil? Can the documentation rig be easily modified to accommodate a range of different tasks?
Technical data comparison and recording settings	Acquisition device Light settings File format Color bit depth Color calibration Recording resolution (PPI) Cell resolution (μm/pixel) Recording dimensions Image size File size	Type of image‐based acquisition technology Light setting (XPL, PPL, OIL, RF and FL) Format types (TIFF, JPEG and others) Maximum bit depth per channel Manual or automatic color calibration Pixels per inch (PPI) Raster resolution in a GIS Maximum size of (pixel × pixel) of recording area Pixel × pixel image size Size (MB) of an uncompressed (TIFF) recording in highest resolution and bit depth.
Image acquisition	General coverage Size/format limitations Thin sections per recording cycle Compound imagery Image acquisition speed Compound imagery process	Size of area recorded of a thin section Maximum recording dimension in the highest resolution Number of output image per recording cycle Numbers of recordings need to create a 60 × 90 mm full‐resolution image Time needed to scan or photograph Postprocessing time taken to combine photos to a single photomosaic
Image quality, in PPL, at different magnifications ×3, ×10, ×30, and ×50	Color/white balance Contrast/dynamic range Texture details Contour sharpness Noise and pixelation Overall image quality	Color reproduction, saturation, and white balance Level of sharpness and blurriness at maximum resolution Amount of texture detail the image conveys Degree of boundary contrast Variation of image density (grains) and degree of pixelation Final ranking of image quality at maximum resolution (PPL)

## 3 IMAGE‐BASED THIN SECTION DOCUMENTATION METHODS

While the five documentation methods selected for evaluation and comparison in this paper do not cover all technical solutions, we would argue that they are representative of the most common techniques currently applied within the field of archaeological micromorphology.

### 3.1 Flatbed scanner

We employ in this paper a relatively inexpensive (approximately €120), consumer grade, A4 photo and slide scanner from Epson (Perfection V370 Photo). Comparable scanners with similar functionality are available from other manufacturers. Typical of most flatbed scanners, including this model, is the moving light source and mirror which directs an image to an image sensor (charge‐coupled device [CCD]) that is located at the lower end of the scanner. The Epson model used here has an effective scanning area of 210 × 297 mm (A4) using reflective light (RL) settings, while the scanning area using TL is limited to 109 × 32.6 mm. Nine 60 × 90 mm thin sections can be scanned at the same time using reflective mode, and a minimum of two scans is thus needed to document the same slide in TL mode (manual stitching required during postprocessing work). A complete RL scan (60 × 90 mm) in the highest resolution possible (7.93 μm/pixel, 8‐bit color depth per channel) results in an uncompressed 460 Mb TIFF file. A complete TL scan (109 × 32.6 mm) in the highest resolution possible (5.29 μm/pixel, 8‐bit color depth per channel) results in an uncompressed 500 Mb TIFF file. Thin sheets of polarizing film can be used for scanning the thin sections in PPL and XPL (Arpin et al., 2002).

### 3.2 Digital film scanner

**Figure 2:**
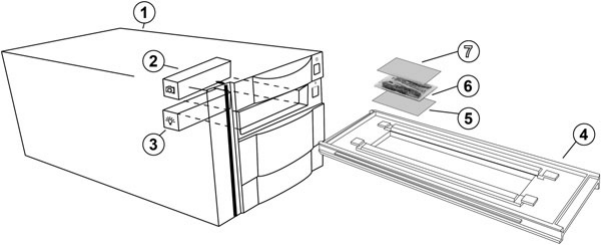
High‐resolution thin section film scanning setup, showing: (1) film scanner chassis; (2) internal optical scanning head with image sensor; (3) light source; (4) scanning strip holder/frame; (5) bottom polarizing sheet; (6) thin section (60 × 92 mm); and (7) upper polarizing sheet

For this study, we use a film scanner from Nikon (2018; Nikon COOLSCAN 8000 ED), but comparable scanners with similar functionality are available from other manufacturers (Figure 2). The Nikon Coolscan 8000 ED is a multiformat film scanner for professional users. It can scan a range of different media, including filmstrips and medical slides. The scanner's ability to process medium format films also allows it to mount thin sections up to 60 × 90 mm by default, and larger slides up to 70 × 120 mm if the frame is physically modified. In the Nikon film scanner, the mirror, light source, and image sensor are fixed, while the frame and medium move.

When scanning with the highest possible resolution (6.35 μm/pixel and 14‐bit color depth per channel) in the largest format available (57 × 83 mm), the Nikon scanner produces an image file of 118 megapixels (13,176 × 8,964 pixels), which corresponds to an uncompressed 250 Mb 8‐bit TIFF file or a 900 MB 14‐bit TIFF‐file (approximately). The scanning time at this resolution would take around 4 min. As with the flatbed scanner, a sheet of polarizing film can be placed below the thin sections in plane‐polarized light (PPL), and a second sheet may be placed above the thin section to produce a scan in cross‐polarized light (XPL).

### 3.3 Stereo zoom microscope (with motorized base)

In this paper, a motorized stereo zoom microscope from Zeiss is used for comparative purposes, but a range of similar microscopes are also available. Our setup comprises a Zeiss Axio Zoom.V16 equipped with a motorized stage (22 × 15 cm) and a Zeiss AxioCam ICc5 camera providing images of 5.0 Megapixels (2,452 × 2,056 pixels) with a 12‐bit color depth per channel (see Supporting Information Video 1). The stereo microscope is fitted with ×0.5 and ×3 objectives and a ×1–16 motorized zoom, resulting in an effective continuous magnification between ×3.5 and ×258. The corresponding field of view is between 52 and 0.9 mm. The setup used has integrated LED lights, enabling observations and thin‐section documentation in reflected light. When different backgrounds are used during image‐capturing (e.g., black, gray, or white), the lights and colors reflect in slightly different ways. Additional equipment for TL, and fluorescent light (FL) combined with various filters are also available but not tested here.

The motorized stage and the stitching software that operates it (Zeiss Zen Core) facilitate the automatic production of the photomosaics in a proprietary Zeiss file format (uncompressed CZI‐file). The CZI format can be edited with the free Zen Lite software package, which also allows for advanced metric measurements. The CZI files are convertible to TIFF‐format using free plug‐ins in combination with third‐party image processing software (e.g., Fiji/ImageJ). Using the motorized stereo microscope at the lowest magnification (×3.5, the number of stitched photos needed to cover the entire thin section is six. Each photo corresponds to a 4,000 × 2,281 pixels TIFF‐file (approximately 21 MB TIFF‐file), and when combined the six stitched photos result in a 300 MB image‐file (uncompressed CZI‐file). Acquisition at medium magnification (×20) requires 130 individual photos to cover the reference thin section and produces a 6.5 GB CZI file. At this magnification, the effective resolution is 3.43 μm/pixel. At low magnification (×3.5) the overall processing time is less than 30 s, and at medium magnification (×20) the scanning and image stitching would take around 10 min.

### 3.4 Macro photography

Although less commonly used than scanners and microscopes, professional DSLR cameras have been reported as a good alternative for the acquisition of high‐resolution thin section imagery (Carpentier & Vandermeulen, 2016; VandenBygaart, Protz, & Duke, 2008). For this paper, we follow the hard‐light configuration presented in Carpentier and Vandermeulen (2016). The macro photography rig comprises a Nikon D800E DSLR camera (a full‐frame camera without anti‐aliasing filter) equipped with an AF‐S Micro Nikkor 60 mm f/2.8 G ED macro lens; all mounted on a macro photography stand. As the photographed thin sections are positioned on a plane under the suspended camera and not inserted into a frame or on top of a scanning surface, there are virtually no physical restrictions regarding the size or shape of the documentation area. Regardless of thin section format, the user can photograph its complete surface using the full resolution of the camera setup (macro photography in 1:1). Photographing at the 1:1 scale of the Nikon D800E allows for a frame of approximately 36 mm by 24 mm, resulting in a 36.3‐megapixel RAW‐file (7,360 by 4,912 pixels and 16‐bit color depth per channel). In practice, eight overlapping photos are needed to cover the complete surface of a 60 × 90 mm thin section, at the highest optical resolution (1:1), resulting in an image size of 18,400 × 12,266 pixels (2.3 GB 8‐bit TIFF‐file) and an effective resolution of 4.8 μm/pixel.

Since the macro photography setup consists of multiple parts (e.g., copy stand, studio flash, glass pane, and mirror), these need installed before images can be acquired. During the photo‐capturing process, great care should be taken to avoid any vibrations; for example, by installing the rig on a solid table, by ensuring a time interval between camera mirror lockup and the actual shot, or by using a remote control to operate the camera without touching it, one can reduce interference from vibrations. It is also important that the camera and the glass pane holding the thin section are both perfectly level. A polarizing film sheet can be placed below the thin section for PPL imagery while another sheet can be held or fixed at the desired angle between the thin section and the lens for XPL pictures. Altering the studio flash angle enables RL photography without moving the thin section.

### 3.5 TL microscope

We employ in this paper a high‐magnification, modular microscope from Zeiss (Axio Imager.A2m), but other brands are available. The Zeiss microscope is a conventional laboratory microscope with a turret that mounts up to seven lenses. In combination with the ×10 eyepieces, the microscope lens used in this study (×5) provides a ×50 magnification. The Axio Imager.A2m is equipped with a digital camera (Zeiss Axio Cam ICc5) which connects to a phototube. The camera model provides an image of 5.0 megapixels (2,452 × 2,056 pixels) with a 12‐bit color depth (per channel) resulting in a 14.5 Mb TIFF‐file. The TL microscope is the only equipment tested in this study that does not allow for a full documentation of the entire thin section, and in our image quality comparison, we therefore confine our evaluation to individual photomicrographs captured at ×50 magnification. The filters used in our study allow for observations in PPL and XPL, but a range of other light settings are also available (e.g., RL or TL).

## 4 RESULTS

### 4.1 Equipment acquisition, setup, and practical use

Several practical aspects concerning acquisition, setup, and efficient use must be taken into consideration before the equipment and documentation methods we have tested in this study can be applied. While a full schematic overview of these aspects is provided in Supporting Information Table A1, we highlight some of the most important differences below.

#### 4.1.1 Availability and costs

The equipment we tested in this study falls into two broad categories: (a) expensive (> €20,000), specialized and custom‐ordered equipment (e.g., stereo zoom microscopes and TL microscopes) and (b) less expensive (< €4,000), commonly available equipment (scanners and DSLR cameras).

#### 4.1.2 Infrastructural requirements

While all the equipment has very similar infrastructural needs (PC, screen, and desk), the macro photography rig requires some additional components, depending on the light source configuration (Carpentier & Vandermeulen, 2016).

#### 4.1.3 Installation and maintenance

While the consumer‐grade flatbed scanner is straightforward to install and maintain, the specialized microscopes require more attention both in terms of installation and long‐term maintenance.

#### 4.1.4 Documentation and preparation procedures

Once the required infrastructure and the equipment are installed for first‐time use, most of the tested equipment can be used without extensive planning or preparation. The exception being the macro photography rig, which most of the time needs to be tuned specifically for each documentation session.

#### 4.1.5 Mobility

While the microscopes used in this study are large and essentially stationary systems, the scanners and the photography rig can easily be transported if necessary.

#### 4.1.6 Versatility

While the film scanner is highly specialized and may only scan translucent media (e.g., film or thin sections), flatbed scanners are slightly more versatile as they are also able to scan and document in reflected (and UV‐light if additional equipment is obtained). Both the DSLR camera and the microscopes can be used as multipurpose gear and are easily modified to facilitate the visual documentation of particular phenomena or material types, whether it is in the lab (microscopes) or in the field (digital camera).

### 4.2 Comparison of technical capabilities and recording settings

When evaluating digital imaging techniques, one of the most relevant aspects is how their technical abilities and image output settings compare; for example, in terms of image resolution, format type, color depth, and file size. In Table 2 we provide an overview of the most relevant technical differences between each of the image‐based thin section documentation methods tested in this paper. The three most important qualities to note are:

1. Light settings, for example, transmitted (TL), reflected (RL), fluorescent (FL);
2. Image format, for example, JPG, TIFF, RAW;
3. Image resolution, for example, cell or pixel size (μm/pixel)

**Table 2:** Comparison of technical capabilities and recording settings

	Flatbed scanner	Film scanner	Macro photography	Stereo zoom microscope	Transmitted light microscope
Acquisition device	Moveable scanning head	Moveable scanning medium	Digital camera (SLR) with macro lens	Digital camera (mounted)	Digital camera (mounted)
Light settings	PPL/XPL/RL	PPL/XPL	PPL/XPL/RL (tested here, FL optional)	RL (tested here; XPL and PPL optional)	PPL/XPL/RL/FL
Format	JPG and TIFF	JPG, TIFF and DNG	JPG, TIFF and RAW	JPG, TIFF and CZI	JPG, TIFF
Color bit depth per channel	8‐bit	14‐bit	16‐bit	12‐bit	12‐bit
Automated color calibration	No	Yes	Yes	Yes	Yes
Resolution (PPI)	3200 PPI (RL) 4800 PPI (TL)	4000 PPI	5200 PPI	96 PPI (single photo) 7405 PPI (mosaic at ×20)	150 DPI (single photo)
Raster cell resolution (μm/pixel)	7.93 (RL) 5.29 (TL)	6.35	4.8 (mosaic)	3.43 μm/pixel (mosaic, 20 ×)	Variable (depending on magnification)
Image dimension (pixels)	9916 × 7008 (RL)13,732 × 4107 (TL)	13,176 × 8964	7360 × 4912 (single)18,400 × 12,266 (mosaic)	2452 × 2056 (single)23,440 × 16,369 (mosaic)	2452 × 2056
Size (Mpx)	56.9	118.1	36.3 (single) 213 (mosaic)	5 (single) 381 (mosaic)	5 (single)
File Size	450 MB (8‐bit TIFF)	950 MB (14‐bit TIFF)	103 MB (single, 16‐bit RAW) 2.3 GB (mosaic, 8‐bit TIFF)	10 MB (single, 16‐bit, TIFF)6.5 GB (mosaic, CZI)	14.5 MB (single, 16‐bit, TIFF)

*Note*. DPI: dots per inch; FL: fluorescent light; SLR: single lens reflex; PPL: plane‐polarized light; PPI: pixels per inch; RL: reflected light; TL: transmitted light; XPL: cross‐polarized light.

The conventional light microscope setups are highly customizable, and a range of different light sources and filters may be applied at additional (and often significant) cost. Whereas both the flatbed scanner and the macro photograph rig can document thin sections in three or more light modes (XPL, PPL, and RL), the film scanner can only operate in two (PPL and XPL). In terms of image format, all tested documentation methods are capable of storing the images in compressed (lossy JPG) and uncompressed (lossless TIFF) file formats, which can display the full detail of the image with no quality loss. Only the film scanner and the DSLR camera can save images in a digital lossless RAW image format that has been minimally processed by the image sensor itself (Adobe's Digital Negative, DNG; and Nikon's RAW format, NEF). RAW image formats often offer extensive image editing capabilities that are not available for other file formats (e.g., through the Adobe Photoshop RAW Converter).

While the motorized stereo zoom microscope only takes single photos with a 96‐DPI resolution, its magnified view at, for example, ×20 provides an effective resolution of 3.43 μm/pixel. This is higher than any of the other thin section‐wide documentation methods tested, such as the flatbed scanner (5.29 μm/pixel), the film scanner (6.35 μm/pixel) and the DSLR camera (4.8 μm/pixel). The image size (megapixel) and file size (MB) is a direct function of image resolution, file compression, and bit‐depth. Consequently, the thin section images with the highest resolution, highest bit‐depth, and lowest compression result in the largest file sizes.

In Table 2 we note that the image file size of the reference thin section (60 × 90 mm) at 3.43 μm/pixel resolution is 6.5 GB (uncompressed CZI file), while the file size of the same thin section at 6.35 μm/pixel resolution is only 0.9 GB (uncompressed TIFF). Yet, a 1 GB TIFF file typically becomes 10–12 times smaller when saved in a high‐resolution compressed format (e.g., JPEG). In our experience the optical qualitative differences between compressed and uncompressed file formats are often minimal. In this respect, however, it is crucial to bear in mind that even though compression may be applied to save storage space, a lossy format might limit the future usefulness of digital thin section images or archives.

### 4.3 Image acquisition

Once the documentation settings are defined, the procedural steps involved in generating thin section‐wide imagery come into play. Each of the methods tested is slightly different and in Table 3 we provide a general overview of the most relevant parameters associated with the image acquisition process. We regard the most relevant parameters to be:

- Thin section documentation coverage (per recording cycle)
- Image acquisition speed (full thin section coverage)
- Postprocessing requirements

**Table 3:** Comparison of image acquisition

	Flatbed scanner	Film Scanner	Macro photography	Stereo zoom microscope	Transmitted light microscope
General coverage per documentation cycle	Whole TS (TL)Partial TS (RL)	Partial TS^a^	Whole TS	Whole TS	Not TS wide
Size/format limitations	210 × 297 mm (RL)109 × 32.6 mm (TL)	57 × 83 mm	None	220 × 150 mm	Not TS wide
Thin sections per shot/scan	9 TS (RL)0.5 TS (TL)	2 TS	1/4 of TS at highest magnification	1	Not TS wide
Compound imagery (mosaic)	No	Yes^a^	Yes	Yes, automated	Limited
Image acquisition speed of full TS coverage	3 min	4 min	1–2 min	Depending on magnification ×3.5:<1 min ×20: approximately 10 min	Instant capture (single image)
Postprocessing required	No	No^b^	Yes (stitching of mosaic)	No	No

*Note*. RL: reflected light; TL: transmitted light; TS: thin section.

^a^ The maximum coverage of the film scanner used in this paper is 57 × 83 mm.

^b^ A thin section that is larger than 57 × 83 mm may be scanned twice and then stitched together afterwards.

A conventional TL microscope with a nonmotorized base is not in practice capable of documenting the reference thin section in its entirety (60 × 90 mm). Some microscopy software solutions do allow for the manual production of photomosaics, but these in‐built features are usually not meant for the production of complete thin section imagery. All other methods tested are capable of recording the entire reference thin section, either by means of automatic (motorized stereo zoom microscope) or manual (DSLR rig) photomosaic production, or through high‐resolution scanning (flatbed scanner and film scanner). Since the TL scanning area of the flatbed scanner (109 × 32.6 mm) is much smaller than that of the RL scanning area (210 × 297 mm), nine thin sections can be scanned during one scanning cycle in TL mode, but only half a thin section may be scanned in RL. It should also be specified that while the film scanner may scan two thin sections at once, the areal coverage (58 × 83 mm) is just below that of our reference thin section (60 × 90 mm). Technically, therefore, the particular film scanner we used does not support a full documentation of an entire 60 × 90 mm thin section. In practice, however, our experience show that the area left out by the film scanner has little impact on the overall visual analysis of the thin section. One should, however, be aware that areal coverage of scanning devices may vary according to the brand, model and light setting.

Image acquisition speed is also quite similar between all tested methods, that is 3–5 min per recording cycle (but up to 10 min with the stereo microscope at ×20 magnification). The exception in this respect is the DSLR camera, which can capture images much faster, that is nine photos in approximately 1–2 min. These nine images, however, need to be stitched together using third‐party software, which adds a variable amount of postprocessing time, depending on the user's experience and the specific software.

### 4.4 Image quality

**Figure 3:**
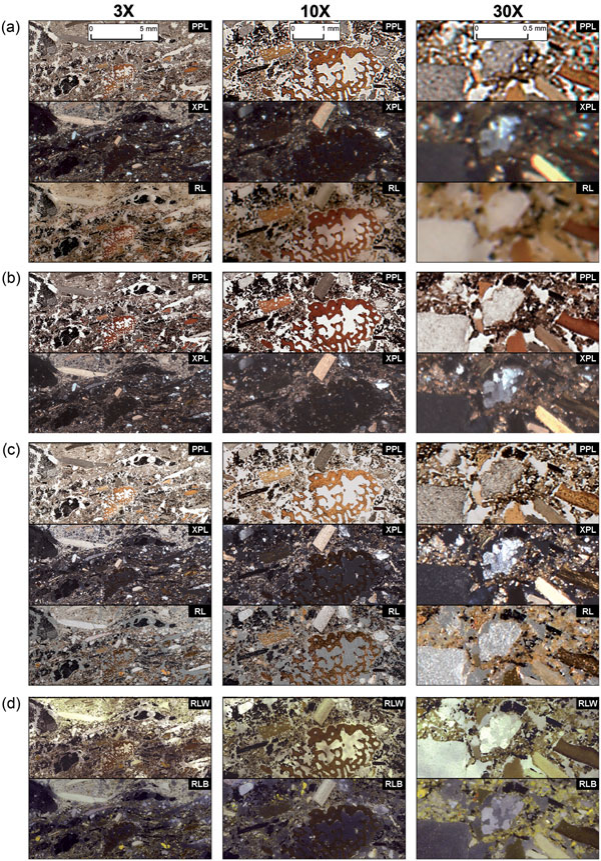
Visual comparison of various thin section wide documentation methods at ×3, ×10, and ×30 magnification. (a) Flatbed scanner, (b) film scanner, (c) DSLR full frame macro photography, and (d) motorized stereo zoom microscope. PPL: plane‐polarized light; RL: reflected light; RLW: reflected light with white background; RLB: reflected light with black background; XPL: cross‐polarized light. See Supporting Information Figures A.11–A.13 for larger versions of these images [Color figure can be viewed at wileyonlinelibrary.com]

**Figure 4:**
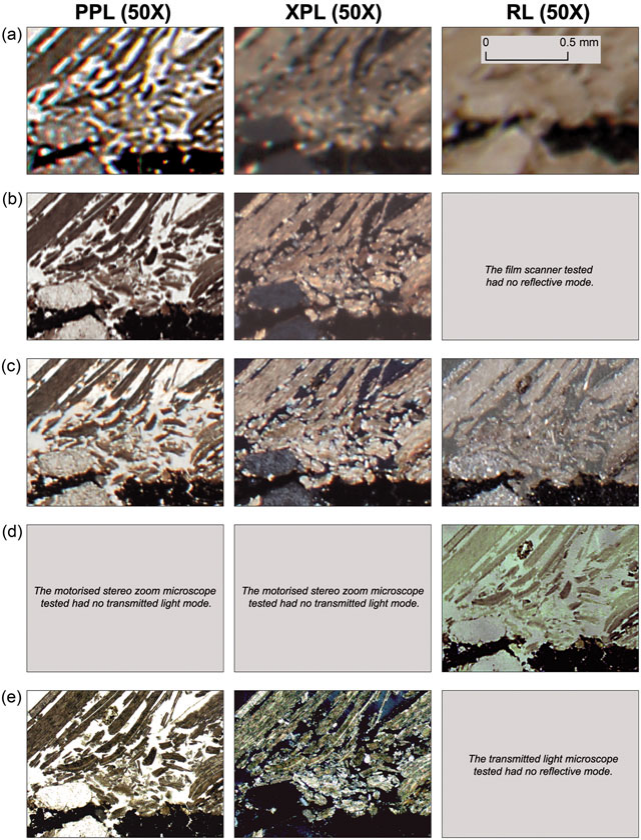
Visual comparison of various thin section‐wide documentation methods at ×50 magnification. (a) Flatbed scanner, (b) film scanner, (c) DSLR full frame macro photography, (d) motorized stereo zoom microscope, and (e) transmitted light microscope. DSLR: digital single lens reflex; PPL: plane‐polarized light; RL: reflected light; XPL: cross‐polarized light [Color figure can be viewed at wileyonlinelibrary.com]

Even though practical use, technical capabilities and image acquisition procedures vary markedly between the different thin section documentation methods, the final output is the same: a high‐resolution rendering of an entire thin section. In Figures 3 and 4 we optically compare the image quality provided by each documentation method, with different light settings and different levels of magnification (×3, ×10, ×30, and ×50; see Supporting Information Figures A11–A13 for larger versions).

We acknowledge that there are at least three critical biases to be aware of when assessing image quality. First, the quality of the output photo may be related to the experience or talent of the photographer or scanning operator. Secondly, the way we perceive images and textures is always somewhat subjective, yet this is also true for observations conducted through the microscope ocular. Thirdly, while in this short contribution we primarily focus on comparing qualitative differences between different documentation techniques, we also recognize that an evaluation of more quantitative aspects, such as spatial accuracy and digital reproducibility, may also be important, particularly if the thin section raster image is to be subjected to spatial or digital image analyses.

To address the first two issues, we have tried to capture photos in the highest quality possible and save these in the highest quality file‐format available (uncompressed), before making direct optical comparisons. Additionally, we compared three parameters that we believe to be most relevant for the evaluation of the overall image quality: texture details, contour sharpness, and level of noise and pixelation. To ensure consistency during our qualitative comparison, each method, light setting, and magnification level was given a relative score between −3 and +3. The lowest score (−3) represents an image quality that is entirely unfit for a basic micromorphological analysis. Higher scores represent an image quality that either is bad (−2), poor (−1), sufficient (0), good (1), very good (2) or excellent (3). In Table 4 we provide an overview of all subscores, as well as a calculation of the average final image quality score. We acknowledge that this quantification might not be perfect, but the overall results correlate well with how these images perform in practice. Our main finds are summarized below:

- **×3 magnification**: All methods provide images with good or very good image quality.
- **×10 magnification**: Most methods provide good image quality, except the flatbed scanner which has a marked decrease in quality (but is still sufficient for most visual analysis).
- **×30 magnification**: While the flatbed scanner provides images that either are poor (PPL) or bad for visual analysis (XPL and RL), the film scanner, macro photography rig, and motorized stereo zoom microscope still create images of sufficient optical quality.
- **×50 magnification**: At this resolution, the flatbed scanner no longer produces images that are meaningful for visual analysis. The film scanner, macro photography rig, and the motorized stereo zoom microscope also show a marked decrease in image quality, in particular for images in XPL, yet some microscopic observations may still be made. At this magnification, the single photomicrographs taken with a conventional TL microscope demonstrate superior image quality.

**Table 4:** Comparison of image quality by documentation method, light setting and level of magnification

	Flatbed scanner			Film scanner		Macro photography			Stereo zoom microscope	Transmitted light microscope	
Level of magnification	PPL	XPL	RL	PPL	XPL	PPL	XPL	RL	RL	XPL	PPL
×3											
×3 Color/white balance*	1*	–	–	2*	–	2*	–	–	–	–	–
×3 Texture details	2	1	1	2	1	2	2	1	1	–	–
×3 Contour sharpness	1	1	1	2	2	2	2	1	2	–	–
×3 Noise and pixelation	1	1	1	2	2	2	2	2	2	–	–
×3 Overall image quality	1.33	1	1	2	1.67	2	2	1.33	1.33	–	–
×10											
×10 Color/white balance*	1*	–	–	2*	–	1*	–	–	–	–	–
×10 Texture details	−1	−1	−1	1	1	2	1	−1	0	–	–
×10 Contour sharpness	1	0	−1	2	1	2	2	2	2	–	–
×10 Noise and pixelation	0	0	0	1	1	1	1	1	1	–	–
×10 Overall image quality	0	0.33	0.67	1.33	1	1.67	1.33	0.67	1.00	–	–
×30											
×30 Color/white balance*	−1*	–	–	2*	–	0*	–	–	–	–	–
×30 Texture details	−1	−2	−2	1	−1	1	0	1	0	–	–
×30 Contour sharpness	−1	−2	−2	−1	−1	−1	−1	−1	1	–	–
×30 Noise and pixelation	−1	−2	−2	0	−1	1	0	0	1	–	–
×30 Overall image quality	−1	−2	−2	0	−1.00	0.33	−0.33	0.00	0.67	–	–
×50											
×50 Color/white balance*	−2*	–	–	0*	–	−1*	–	–	–	2*	–
×50 Texture details	−2	−3	−3	−1	−2	−1	−2	−2	−1	3	2
×50 Contour sharpness	−2	−3	−3	−2	−2	−1	−1	−2	0	3	3
×50 Noise and pixelation	−2	−3	−3	−1	−2	−1	−2	−2	−1	2	2
×50 Overall image quality	−2	−3	−3	−1	−2	−1	−1.67	−2	−0.67	2.5	2.33

* The number score provided for “color and white balance” reflects our evaluation of raw and unprocessed image outputs. Because color and white balance may be easily corrected during postprocessing procedures, these scores are not included in the calculation of “Overall image quality score.”

It should be noted that the image mosaic produced by the stereo zoom microscope was made by multiple photomicrographs captured at ×20 magnification. These images could have been captured at higher magnification, and thus provided better optical quality, but at the cost of lower recording speed and considerable larger file size.

## 5 ANALYTICAL AND PRACTICAL APPLICATIONS

Goldberg and Aldeias (2018) have argued that as a subdiscipline within the broader field of geoarchaeology, archaeological micromorphology is still an underutilized, underestimated, and sometimes misunderstood analytical technique, particularly amongst field archaeologists. They have suggested that one of the main reasons for this is an overemphasis on text‐based or semiquantiative descriptions of thin sections and a general lack of more intuitive and instructive graphics, which can be contextualized and investigated on multiple scales. While we largely agree with the view of Goldberg and Aldeias (2018), we would also like to add that the lack of necessary laboratory infrastructure in many archaeological departments (e.g., lack of petrographic microscopes) combined with an often limited access to comparative or educational thin section collections may also be restricting factors for students and researchers wanting to employ archaeological micromorphology for their sites or projects.

Considering the results of our method comparison (Figures 3 and 4, and Supporting Information Figures A1–A13), we think that by adopting a high‐resolution digital documentation practice, many of the concerns and limitations posed by Goldberg and Aldeias (2018) and others could be addressed. In Table 5, therefore, we present an overview of the most important benefits of producing and working with digital images of entire archaeological thin sections. In this table, we have identified a range of practical and analytical domains in which archaeological micromorphology may significantly improve or expand if incorporated into the analytical and practical workflow. While it is beyond the scope of this paper to discuss all these aspects in detail, we would like to highlight the usefulness and importance of acquiring high‐resolution digital images of entire thin sections by emphasizing five general areas: (a) logistics, infrastructure and efficiency; (b) multiscalar and micro‐contextual analysis; (c) accessibility, education, training; (d) scientific communication; and (e) archiving and collection management.

**Table 5:** Overview of analytical and practical benefits of producing and working with high‐resolution digital images of entire archaeological thin sections

Implications	For	Why
Practical	Efficiency	Thin section analyses low to medium magnification conducted directly on a computer screen are far more efficient and comfortable than looking through the ocular of a microscope. Less time may be spent on basic material observations, and more time can be dedicated to more complex material or microscopic relationships.
	Logistics	Considerable microscope time can be freed when much of the low‐to‐medium‐scale investigations (×30) are moved to a digital investigative platform (e.g., a computer screen).
	Organizing and archiving	Having a digital archive of high‐resolution thin section images makes it easier to digitally organize thin sections by date, projects or topics, etc. A digital approach to sample management may be applied on small‐scale collections (e.g., individual projects) or it may lay the foundation of entire working group archives or even globally accessible repositories. Also, thin sections of glass are fragile, may deteriorate over time or may get destroyed by user‐related mistakes. A digital archive of high‐resolution thin section images is not subject to these degradational issues.
	Collaboration	A digitized thin section may be shared and coanalyzed with colleagues, either through conventional file‐sharing or through collaborative systems (e.g., over the Internet) that allows for real‐time editing and analysis of graphic material.
	Education	Once a thin section is digitally documented, it can easily be used for educational purposes, for example, as visual reference in a lecture or through more interactive exercises where students are given digital datasets instead of or in addition to accessing physical thin sections training sets.
	Presentations	Documenting entire thin sections allows you to effortlessly select the part of it that is most relevant, and then save the chosen area in a customized format (size) at the most appropriate level of magnification. Consequently, more diverse and complex visual thin sections presentations can be made and used in posters, presentations or in academic journals.
Analytical	Field of view	Images of whole thin sections represent a superior field of view compared with microscope micrographs, and in high resolution they allow for more accurate and coherent multiscale observations of complex relationship and features and their spatial distribution across the whole thin section.
	Multiscalar analysis	Because one image can visualize the same thin section at multiple levels of magnification, one can seamlessly investigate the occurrence and distribution of mesoscale to microscale relationships; either in image editing software or in a GIS environment (see below).
	Image analysis	Multiple types of image analyses ought to benefit from thin section‐wide documentation, including simple raster manipulation, more advanced quantitative techniques and machine learning approaches.
	Spatial analysis	Georeferenced thin section documentation allows for direct metric measurements on the thin section images themselves (as in microscopy software), but it also provides a better overview of the distribution of sedimentary material and microstructure, as these may be accurately tracked over partial or entire thin sections, or even across multiple thin sections (Haaland et al., 2017).
	Multidisciplinary integration of data	Once a thin section is visually documented and spatially referenced within the archaeological site, all micromorphological observations and interpretations may be directly contextualized with other georeferenced datasets, allowing for a more intuitive and robust integration of multidisciplinary data collected at multiple scales and with different methods. This includes the integration of other microanalytical techniques applied directly on the thin sections, such as elemental mapping (micro‐XRF) or microscopic infrared spectroscopy (micro‐FTIR).

### 5.1 Logistics, infrastructure, and analytical efficiency

By moving much of the low‐to‐medium scale micromorphological investigations (× <30 magnification) over to a digital investigative platform (i.e., a computer screen), time spent on microscopy can be freed and allocated more exclusively to medium and high‐resolution analysis. Given that the prices for advanced film scanners or a macro photography setup are only a fraction (< €4,000) of the cost of conventional TL microscopes (> €20,000), investing in them may potentially reduce some of the infrastructural costs and logistical requirements for conducting basic petrographic analysis; in particular for archaeological departments that have many students but few TL microscopes available.

In terms of analytical efficiency, conducting parts of the qualitative thin section analyses directly on a larger computer screen has proven far more efficient and comfortable than looking through the ocular of a microscope. Our own experience indicates that the time needed to conduct basic material observations is considerably reduced, and that more time can therefore be dedicated to evaluating more complex material and microscopic relationships at the microscope. Although it should also be emphasized that at present, digital imaging technology is not capable of substituting all settings or analytical configurations offered by conventional petrographic microscopy. For specific light settings, optical alignments (e.g., the use of a Bertrand lens) or to focus on very small features at high magnification (e.g., spherulites) a petrographic light microscope is still, for the moment, the only viable option.

### 5.2 Multiscalar and microcontextual analysis

With high‐resolution thin section‐wide documentation being applied, more comprehensive digital image analysis may be conducted; allowing for novel and more powerful ways of converting thin section raster data into analytically meaningful figures, maps or graphs. For example, once the entire thin section is digitized one can start exploring, documenting, and analyzing complex relationships and features that would be difficult to appreciate through spatially constrained microphotographs taken with a conventional petrographic microscope setup.

Multiple types of image analyses ought to benefit from this, such as basic raster manipulation and various types of quantitative image analyses (Goldberg & Macphail, 2006) to more sophisticated and novel approaches, such as machine learning (Budennyy et al., 2017; Ross, Fueten, & Yashkir, 2001). The power of *digital micromorphology* becomes particularly evident when thin section‐wide imagery is implemented into georeferenced investigative frameworks, where in combination with other archaeological datasets, they are capable of bridging the gap between microscale and macroscale investigations of archaeological contexts (Fisher et al., 2015; Haaland, Friesem, Miller, & Henshilwood, 2017; Karkanas et al., 2015).

### 5.3 Accessibility, education, and training

Once a thin section is digitally documented, it can be digitally shared with a much larger audience than is the case with glass thin sections. This may drastically increase accessibility to physical thin section collections. Apart from promoting professional collaboration, easier access to important thin section collections may also positively affect training and education in archaeological micromorphology. Digital thin section image collections could, for example, provide visual references for lectures or facilitate more interactive exercises where students are given digital datasets instead of, or in addition to, physical thin section training sets. Not only would public digital thin section training sets allow students to gain experience in analyzing a much larger range of archaeological contexts, but they could also serve as an intracommunity and consensus‐based training ground in which material identification is collectively studied and defined. Such a system may also address several of the concerns raised by Shahack‐Gross (2016) related to differences in training and the general lack of self‐evaluation, and hence might help to improve the accuracy of qualitative micromorphological data production in the long term.

### 5.4 Scientific communication

The usefulness of high‐resolution thin section images becomes even more evident considering the medium through which most geoarchaeological research is communicated: the academic journal. While most journals are optimized for scientific communication through text, they are not ideal for the dissemination of large visual datasets. This is not only a disadvantage but may hinder methodological transparency, scientific reproducibility and ultimately the credibility of archaeological micromorphology as a scientific discipline. The availability of complete empirical datasets is especially crucial for the ability of peers to assess the validity of microscale observations as well as higher‐level interpretations (e.g., site formation or landscape changes). The systematic visual documentation of entire thin sections does not necessarily solve this issue in itself, but once a thin section is digitized in high resolution, it becomes easier to produce relevant image data of high quality, in different light settings at different scales and in combination with other archaeological datasets (Fisher et al., 2015; Haaland et al., 2017; Karkanas et al., 2015). Digital thin section datasets can be shared more easily as electronic collections, and thus promote inter‐laboratory collaboration.

### 5.5 Archiving and collection management

Having a digital archive of high‐resolution thin section images makes it easier to digitally organize thin sections by date, projects, or topics. A digital approach to thin section management may be applied to small‐scale collections (e.g., individual projects) or it may lay the foundation for entire working group archives or even globally accessible repositories. If thin section imagery is properly archived and stored as high‐resolution digital collections, the optical quality of such digital records does not degrade over time, and it is not prone to cracking and weathering, as is sometimes the case with physical thin sections made of glass. Thus, in theory, the digital version of the thin section could outlast the physical version. In practice, however, the digital archiving system used needs to be carefully designed to ensure that all relevant information is stored with appropriate and consistent quality, in accessible file types and organized following standards and protocols that ensure universal access and long‐term preservation of digital data (Brin, 2013).

## 6 CONCLUSION

In this paper, we compared and evaluated five common image‐based methods for documenting archaeological thin sections in high‐resolution: a flatbed scanner, a film scanner, a macro photography rig, and conventional stereo and light microscopes. Based on our comparison results, we conclude that advances in digital imaging technology now allow for easy, fast, and high‐quality visual recording of entire thin sections, in multiple light settings and up to at least ×30 magnification using relatively inexpensive recording equipment, such as film scanners and DSLR cameras.

In our discussion we argue that there are many benefits of acquiring high‐resolution digital images of entire thin sections. First, a large part of the otherwise time‐consuming and basic optical analyses that previously were carried in front of a TL microscope, can in many cases be replaced by visual inspections of digital imagery on a large computer screen. Digitizing the micromorphological practice in this way may not only improve the efficiency and consistency of conducting basic, low‐to‐medium magnification thin section observations, but it may also allow qualitative observations to be more intuitively and accurately visualized and analyzed across entire thin sections in ways that simply are not possible using only single photomicrographs. Furthermore, once a thin section is digitally recorded and digitally archived, it can also be remotely and digitally shared and collectively discussed and analyzed on a level of consistency and transparency that most physical thin section collections would hardly reach. The establishment of openly accessible digital thin section archives or repositories might consequently lead to increased scientific reproducibility and inter‐laboratory communication, as well as lay the foundations for more consensus‐based educational training grounds, in which students of archaeological micromorphology may gain more coherent analytical experience.

Considering all of the direct and potential benefits of digital thin section imagery, we hope that the discussion of documentation methods presented in this study will stimulate future archaeological micromorphologists to produce and share more comprehensive visual datasets, if not in the scientific papers themselves, then in digital online appendices or openly accessible repositories. A complete data set of uncompressed, high‐resolution thin section images generated from this study is available for download at www.geoarchaeology.info/digital_thin_sections.

## Supporting information

Supporting information

Supporting information
