# Supplementary material for: Documenting archaeological thin sections in high‐resolution: A comparison of methods and discussion of applications
Source: Geoarchaeology. 2018 Nov 13;34(1):100–14. doi: 10.1002/gea.21706 (PMC6358115; doi:10.1002/gea.21706)
Supplement: Supplementary file 1 — Supporting information [file GEA-34-100-s001.docx]

**Supplemental Information**

**Documenting archaeological thin sections in high-resolution: a comparison of methods and discussion of analytical applications**

Magnus M. Haaland ^1,2*^

Matthias Czechowski ^3,4^

Frank Carpentier ^5^

Mathieu Lejay ^6^

Bruno Vandermeulen ^7^

^1^ Institute for Archaeology, History, Culture and Religious Studies, University of Bergen, Øysteinsgate 1, PO Box, N-5020, Bergen, Norway

^2^ Centre for Early Sapiens Behaviour (SapienCE), University of Bergen, Sydensplassen 12/13, N-5007 Bergen, Norway

^3^ Institute for Archaeological Sciences, University of Tübingen, Rümelinstr. 23, 72070 Tübingen, Germany

^4^ Senckenberg Center for Human Evolution and Palaeoenvironment, Sigwartstraße 10, 72076 Tübingen, Germany

^5^ Department of Archaeology, KU Leuven, Leuven, Belgium

^6^ TRACES Laboratory – UMR 5608, University of Toulouse 2 Jean Jaurès, Toulouse, France

^7^ ULS Digitisation and Document Delivery, KU Leuven, Leuven, Belgium

***Corresponding author,**

Magnus M. Haaland

E-mail: magnus.haaland@uib.no

**This supplemental information includes:**

Table A.1

Figure A.1 – A.13

A complete dataset of uncompressed, high-resolution thin section images generated from this study will be available for download at: www.geoarchaeology.info/digital_thin_sections.

Supplemental Table 1 Comparison table of: (A) Equipment acquisition, cost, infrastructural requirements and maintenance level; (B) Installation and preparation time; and (C) practical use. See table 1 for explanation of each variable.

|  |  | **Flatbed**  **scanner** | **Film**  **scanner** | **Macro**  **photography** | **Stereo-**  **microscope** | **Transmitted**  **light microscope** |
| --- | --- | --- | --- | --- | --- | --- |
| **A** | Availability | Common | Common | Common | Special | Special |
|  | Cost | €50-200 | €1000 – 3000 | €2000 – 4000 | >€20 000 | >€20 000 |
|  | Required infrastructure | PC, screen, desk | PC, screen, desk | Photo stand, desk, light-source, camera, lens | PC, screen, desk, microscope | PC, screen, desk, microscope |
|  | Maintenance level | Low | Medium | Medium | Medium/high | Medium/high |
| **B** | Installation duration (first time) | Low | Low | Medium | High | High |
|  | Preparation complexity | Low | Medium | High | Low | Low |
|  | Preparation time before routine use | 1 minute | 5 minutes | 10-15 minutes | 1 minute | 1 minute |
|  | Preparation time per documentation cycle | Low | Medium | Medium/low | Low | Low |
| **C** | Mobility | Medium | Medium | High/medium | Low | Low |
|  | Use with oil | Yes | Yes | Yes | Yes | Yes |
|  | Versatility | Medium | Low | High | High | High |


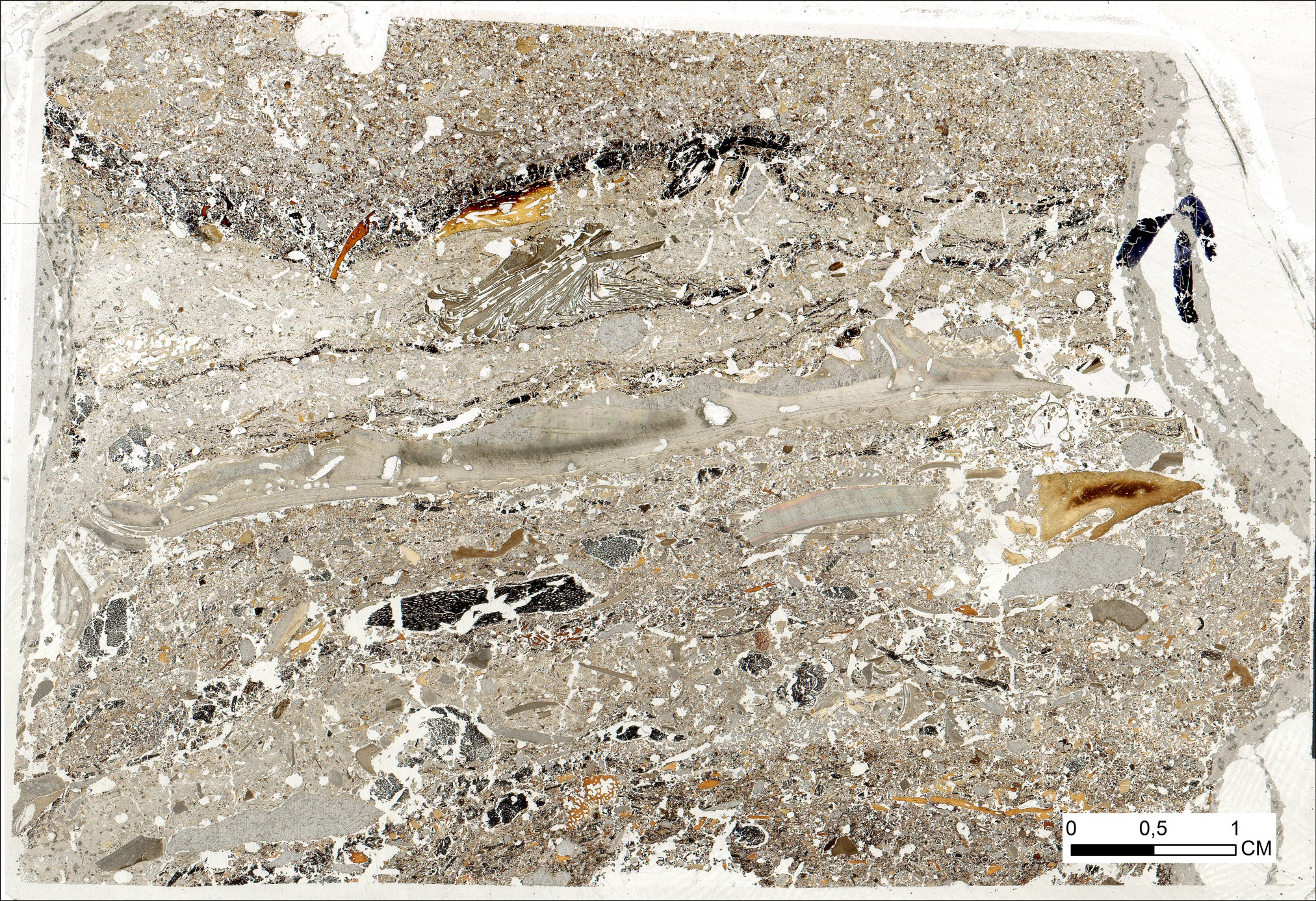


Fig. A 1 Flatbed scan (PPL) of the reference thin section (recorded in 4800 DPI, displayed here in 600 DPI).


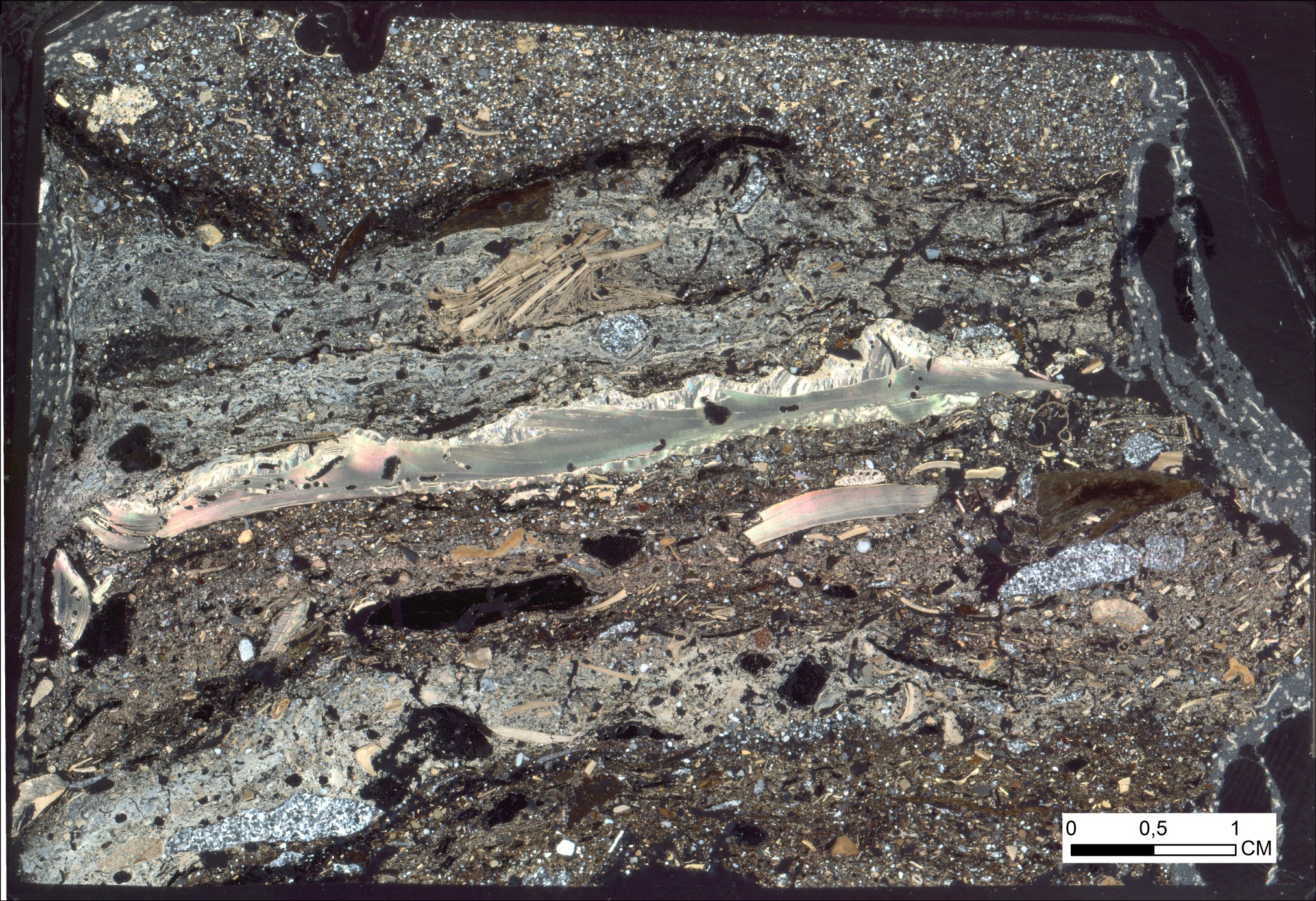


Fig. A 2 Flatbed scan (XPL) of the reference thin section (recorded in 4800 DPI, displayed here in 600 DPI).


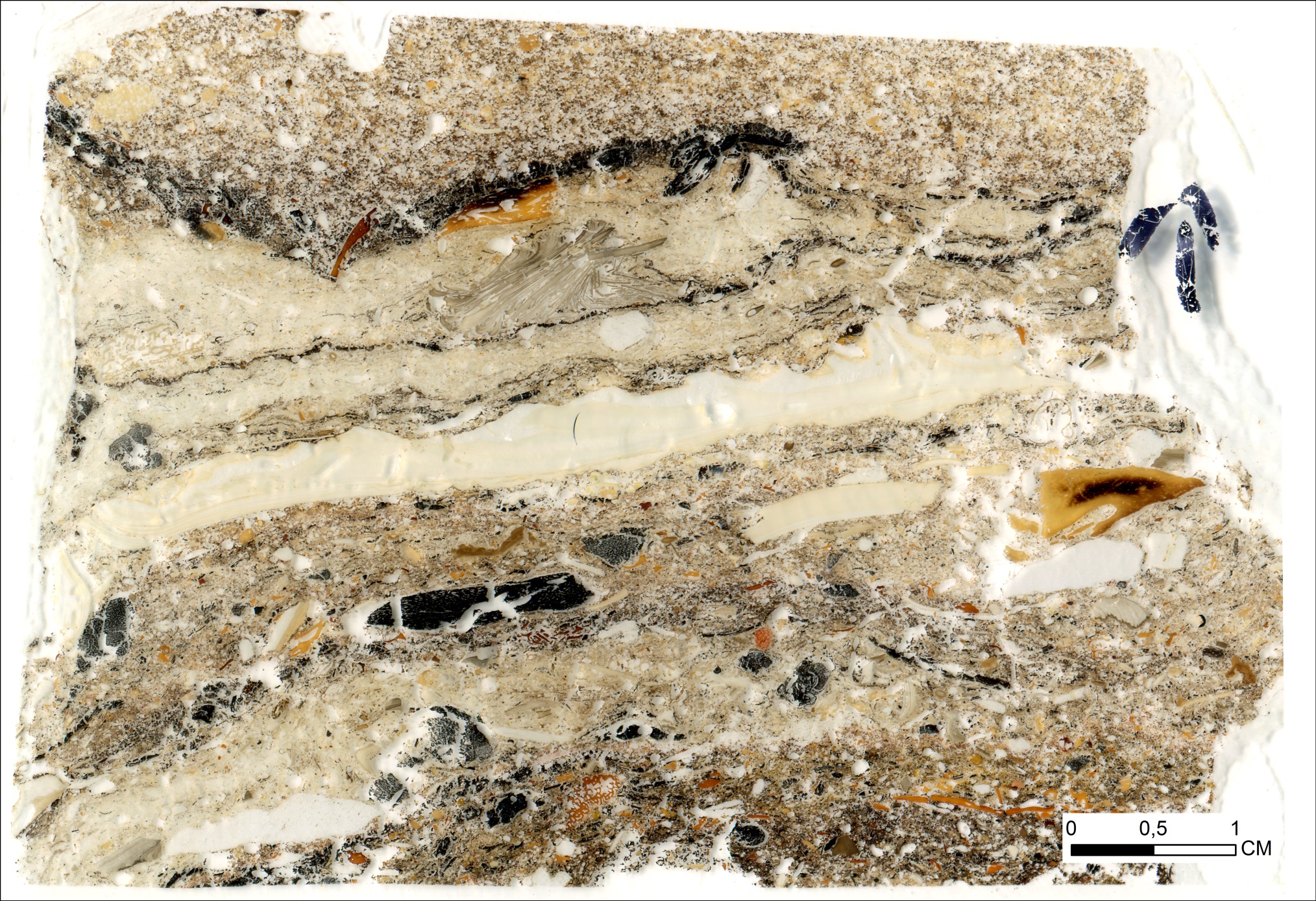


Fig. A 3 Flatbed scan (L) of the reference thin section (recorded in 3200 DPI, displayed here in 600 DPI).


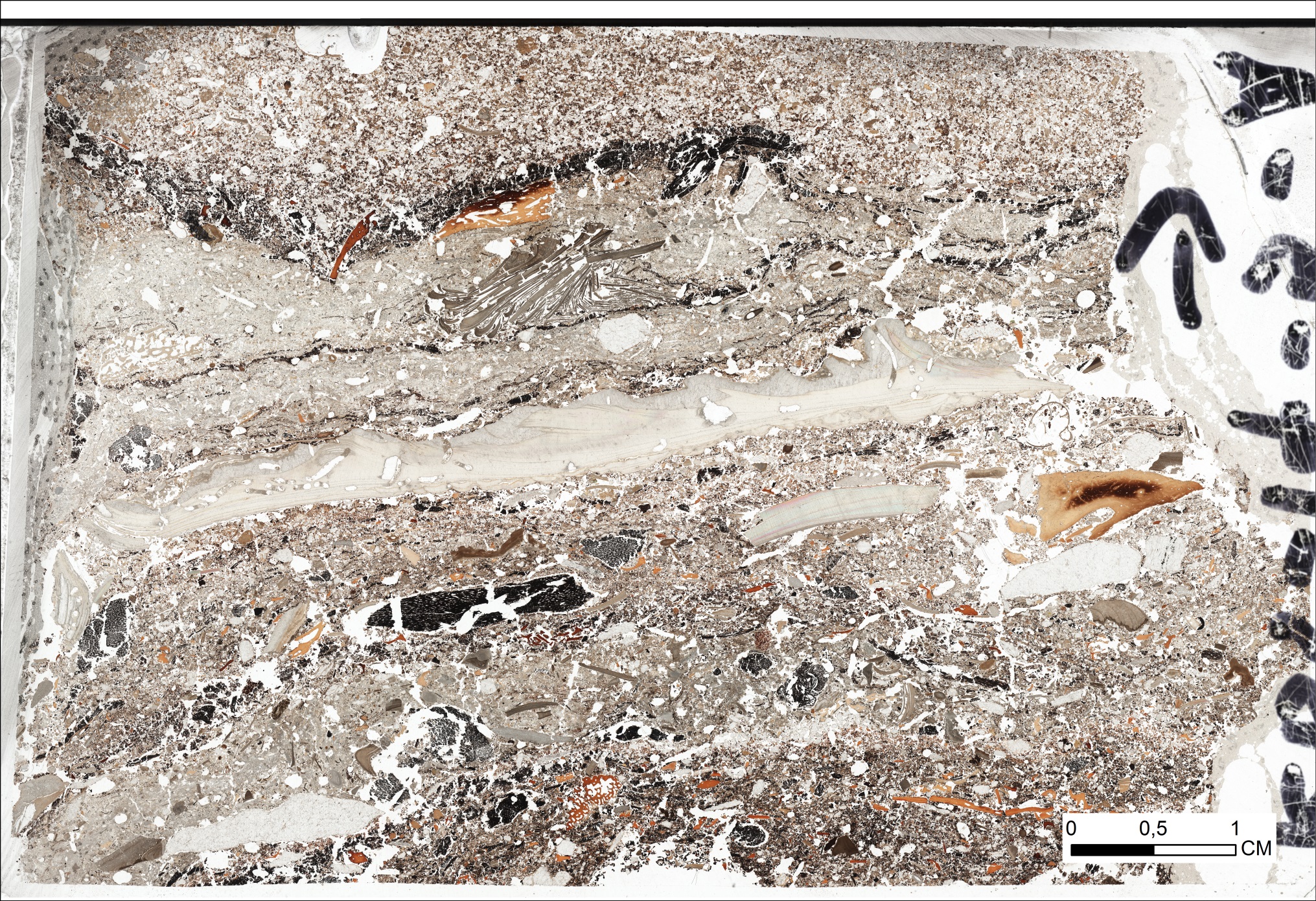


Fig. A 4 Film scan (PPL) of the reference thin section (recorded in 4,000 DPI, displayed here in 600 DPI).


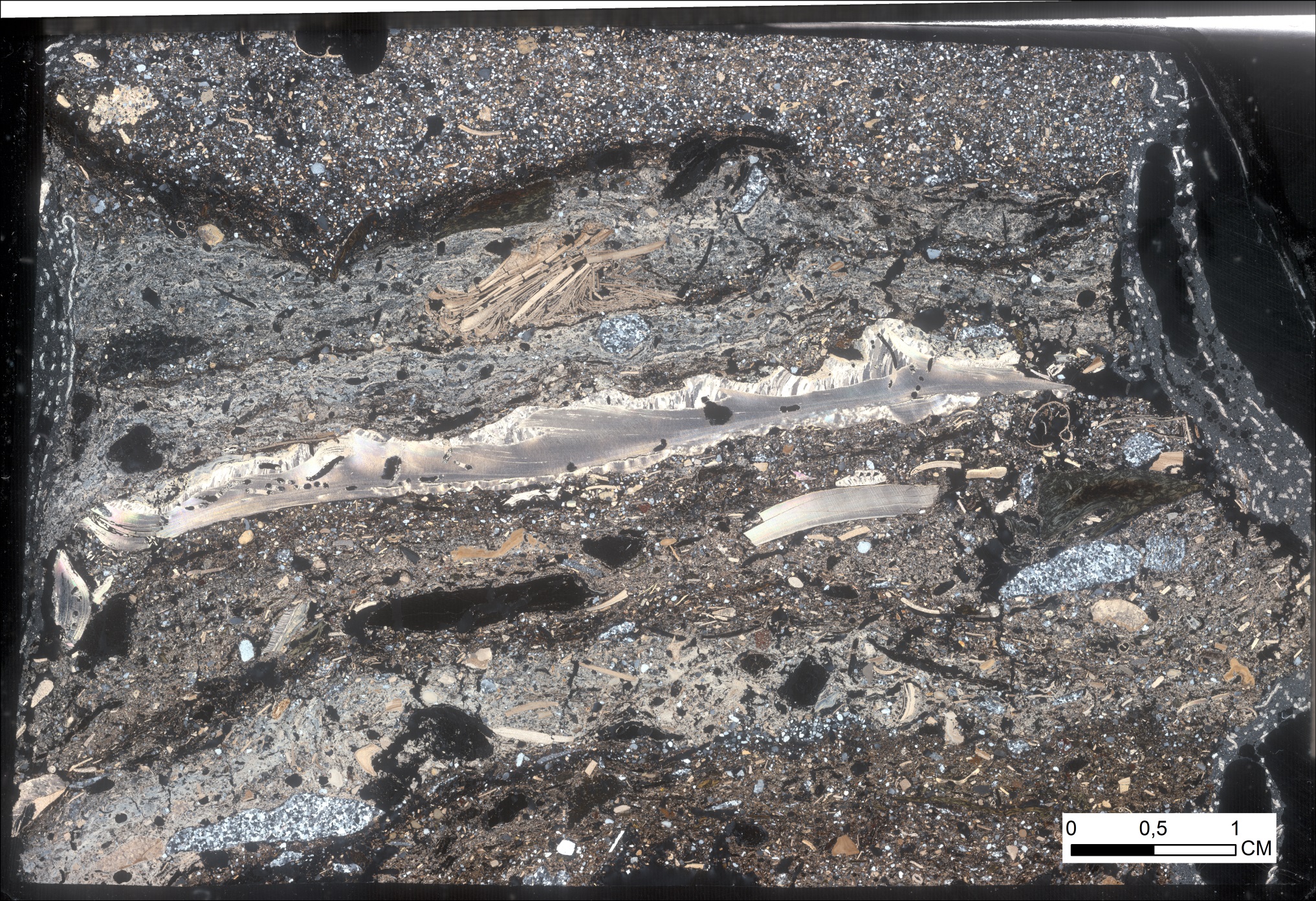


Fig. A 5 Film scan (XPL) of the reference thin section (recorded in 4000 DPI, displayed here in 600 DPI).


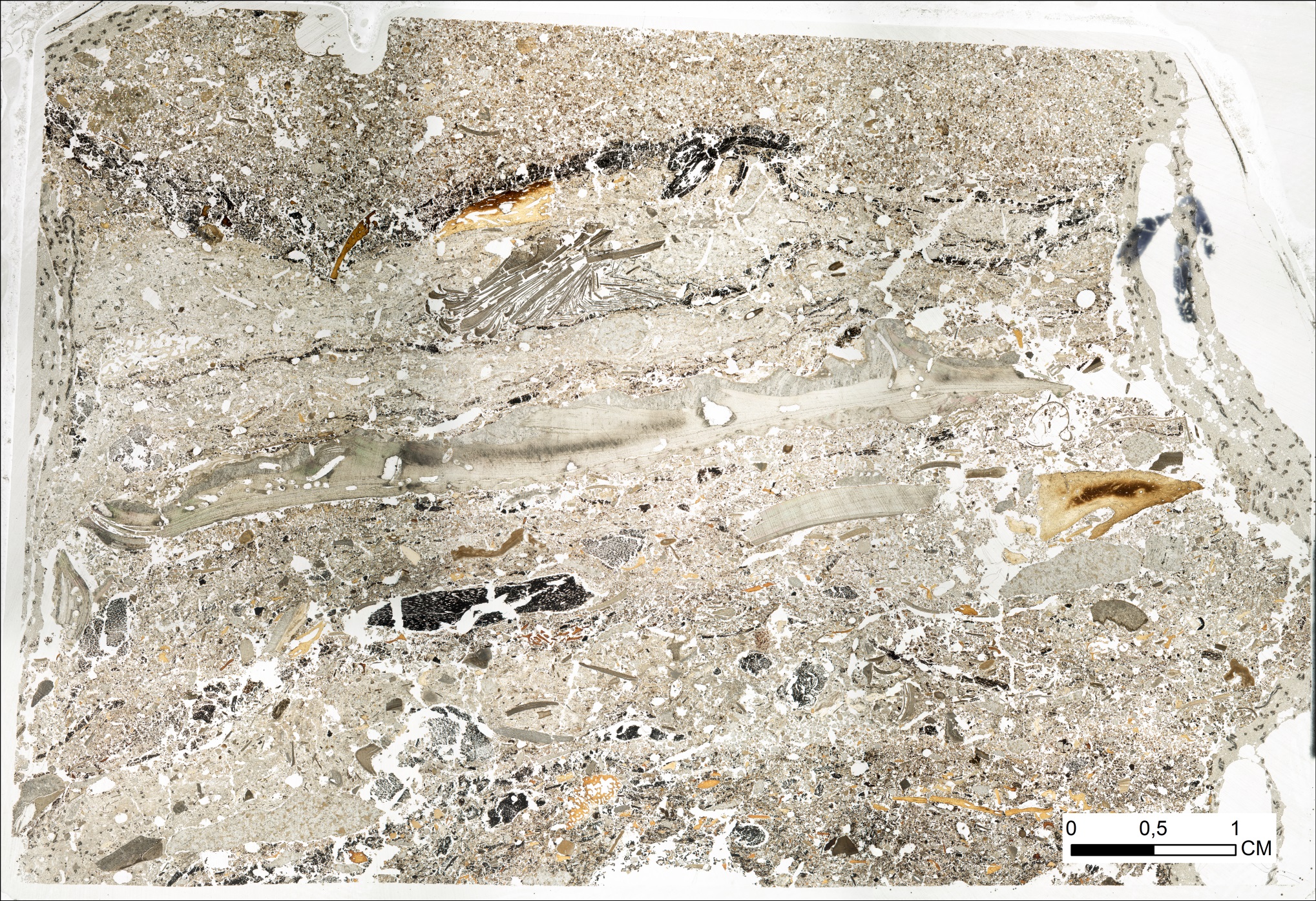


Fig. A 6 Macro photograph captured with a DSLR camera (PPL) of the reference thin section (recorded in 4200 DPI, displayed here in 600 DPI).


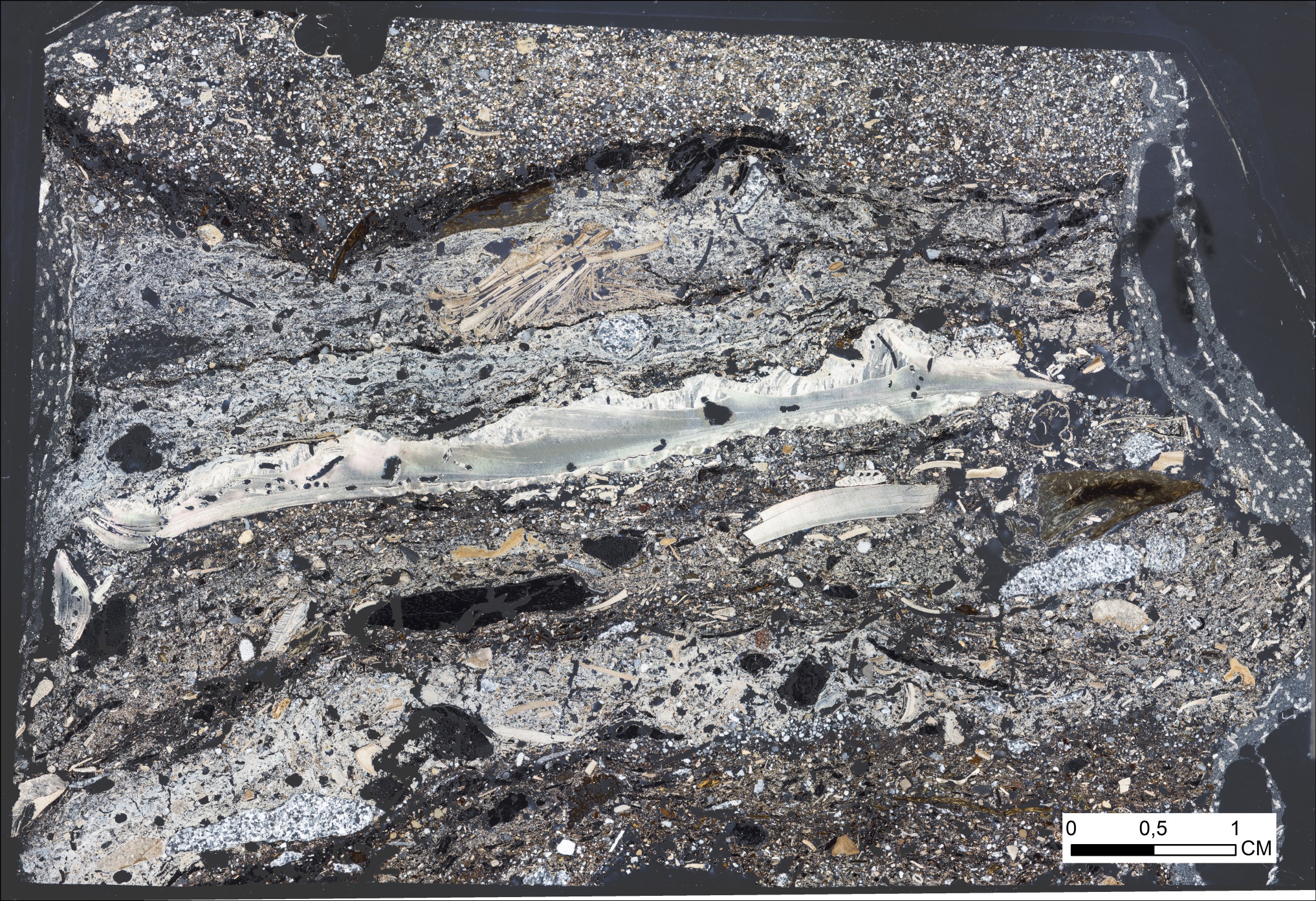


Fig. A 7 Macro photograph captured with a DSLR camera (PPL) of the reference thin section (recorded in 4200 DPI, displayed here in 600 DPI).


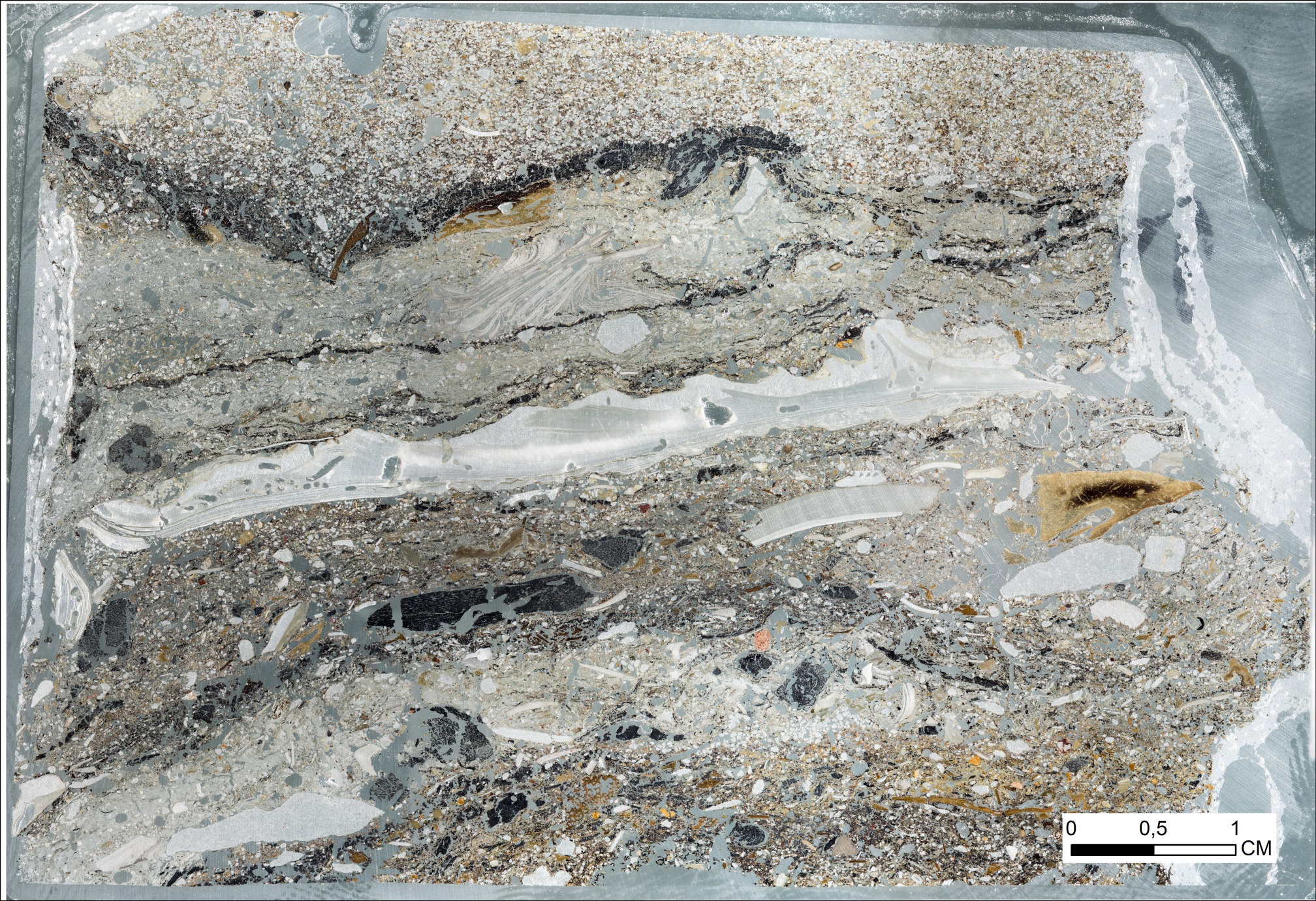


Fig. A 8 Macro photograph captured with a DSLR camera (RL) of the reference thin section (recorded in 5200 DPI, displayed here in 600 DPI).


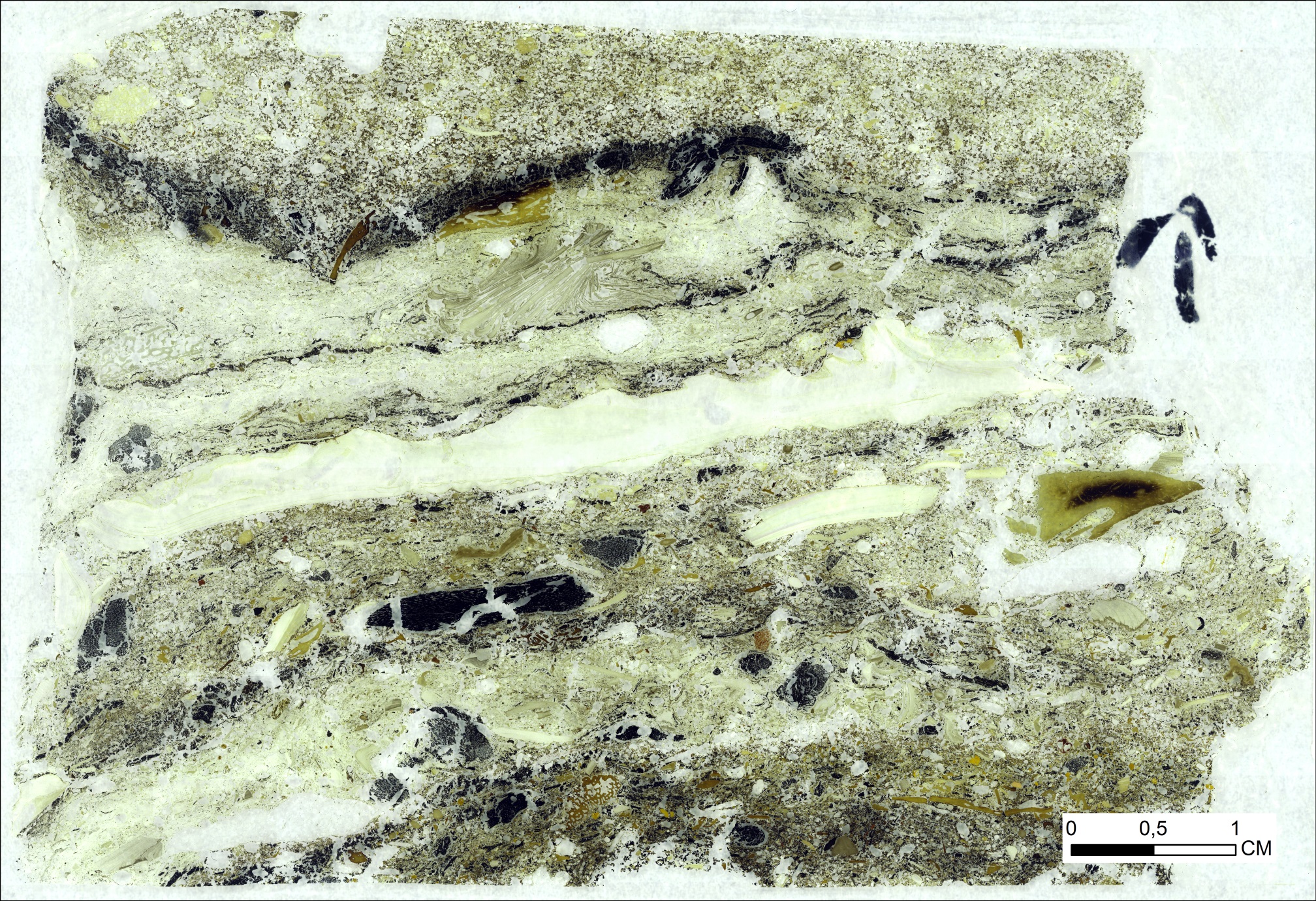


Fig. A 9 Stereo zoom microscope mosaic (RL mode with white background) of the reference thin section (recorded in 7405 DPI, displayed here in 600 DPI).


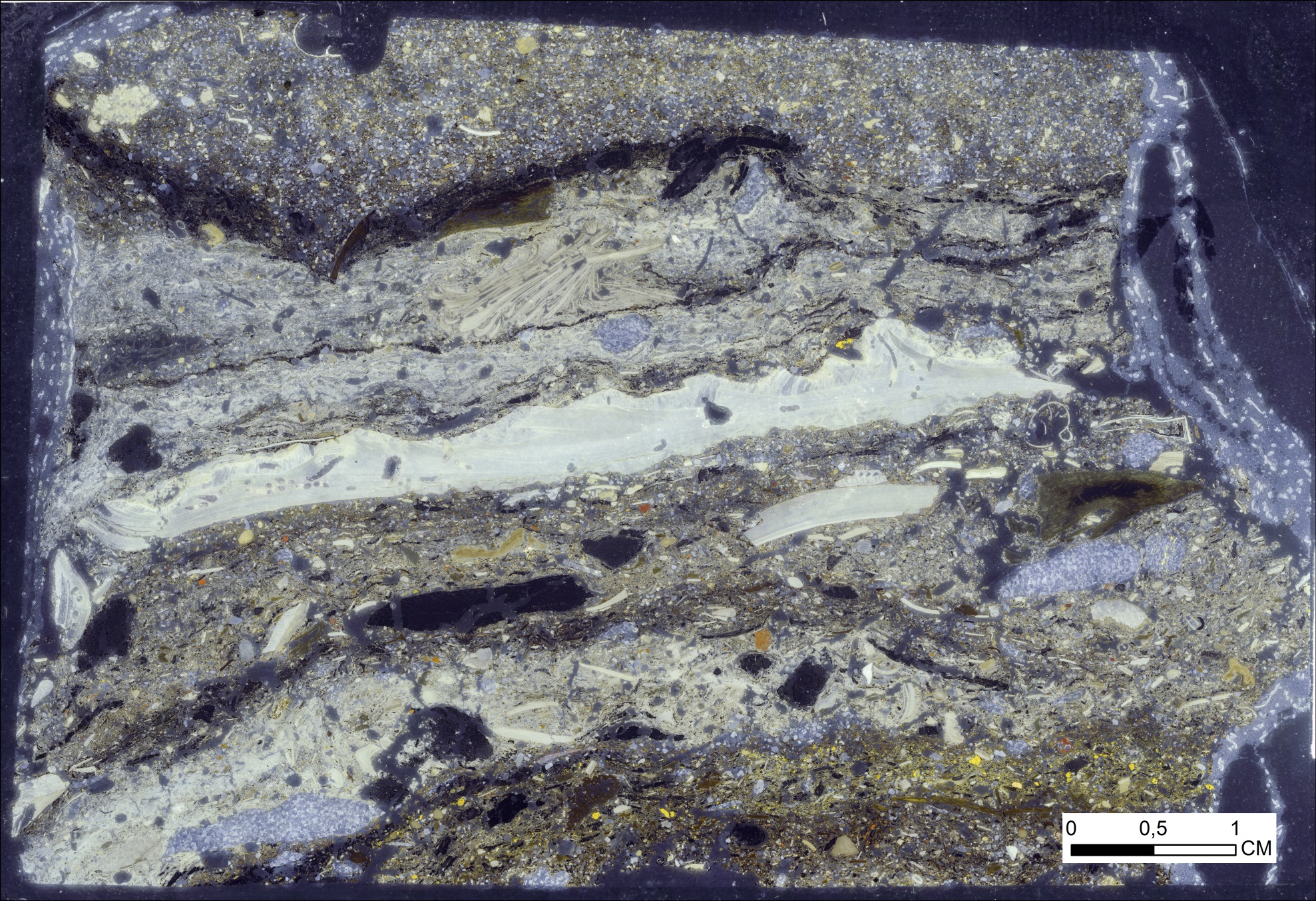


Fig. A 10 Stereo zoom microscope mosaic RL mode with black background) of the reference thin section (recorded in 7,405 DPI, displayed here in 600 DPI).


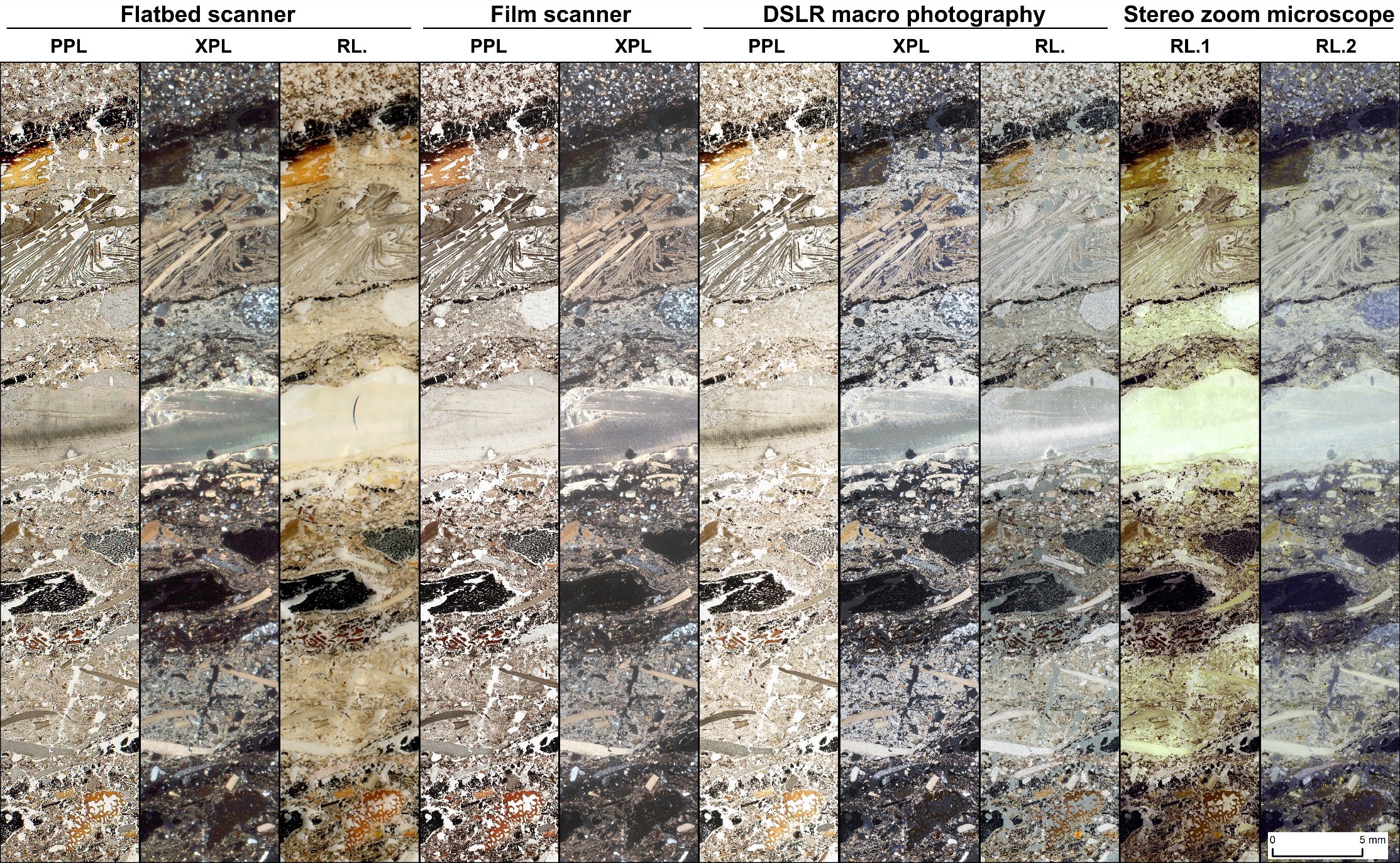


Fig. A 11 Comparison of all thin section documentation methods (3x). RL1: Reflected Mode 1: White background. RL2: Reflected mode 2: black background.


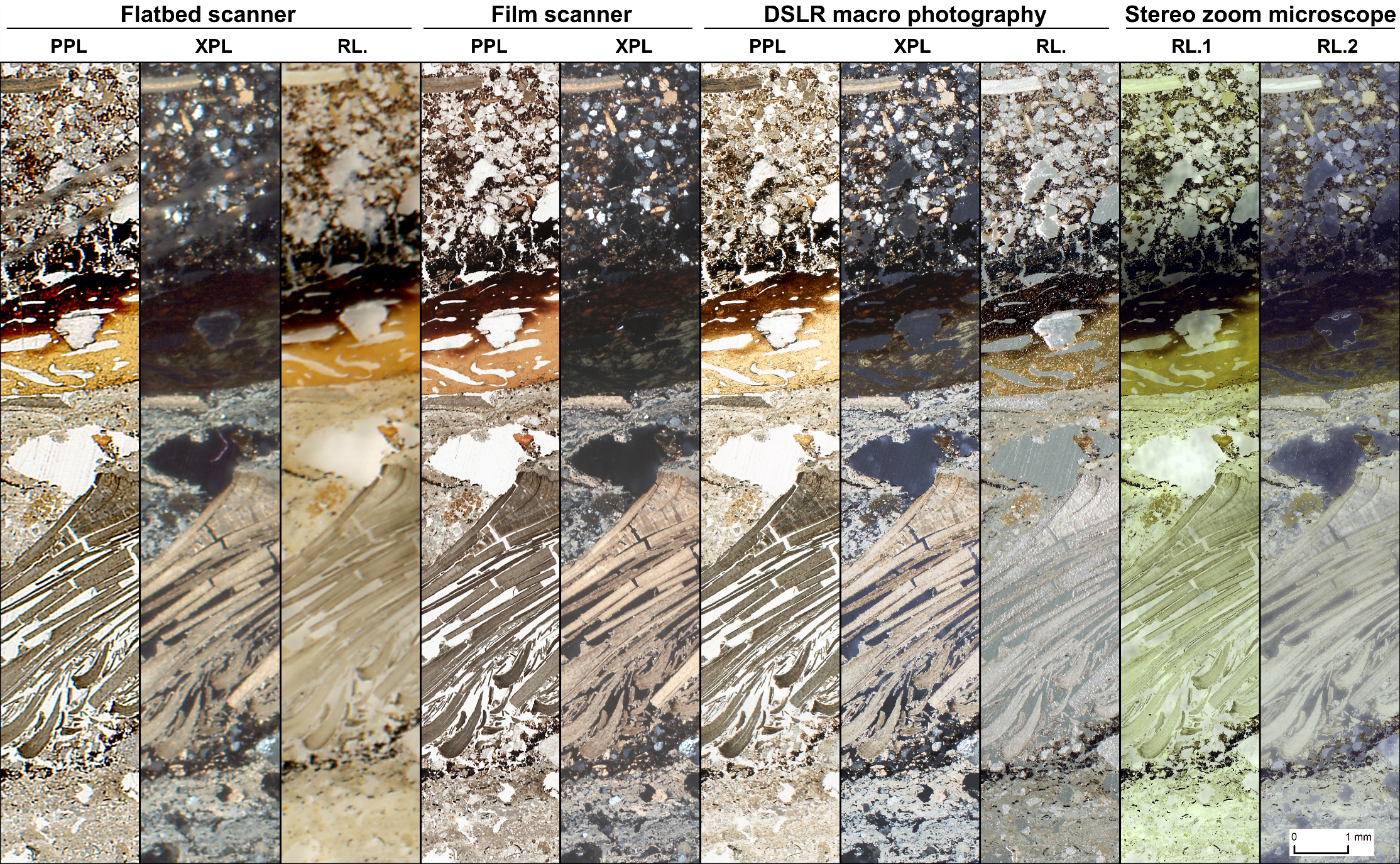


Fig. A 12 Comparison of all thin section documentation methods (10x). RL1: Reflected Mode 1: White background. RL2: Reflected mode 2: black background.


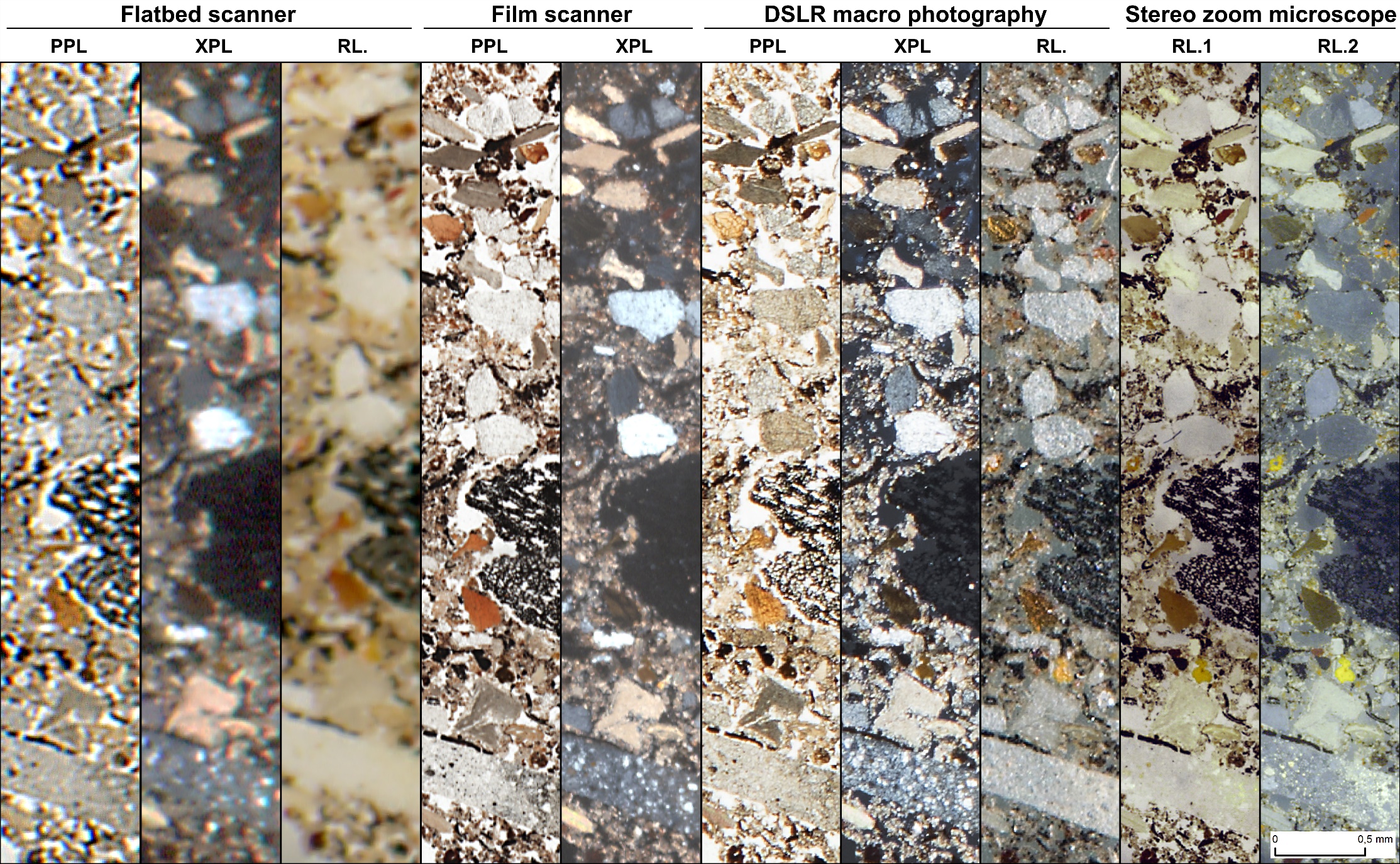


Fig. A 13 Comparison of all thin section documentation methods (3x). RL1: Reflected Mode 1: White background. RL2: Reflected mode 2: black background.
